# Combining Positive Matrix Factorization and Radiocarbon Measurements for Source Apportionment of PM_2.5_ from a National Background Site in North China

**DOI:** 10.1038/s41598-017-10762-8

**Published:** 2017-09-06

**Authors:** Xiaoping Wang, Zheng Zong, Chongguo Tian, Yingjun Chen, Chunling Luo, Jun Li, Gan Zhang, Yongming Luo

**Affiliations:** 1 State Key Laboratory of Organic Geochemistry, Guangzhou Institute of Geochemistry, Chinese Academy of Sciences, Guangzhou, 510640 China; 2 Key Laboratory of Coastal Zone Environmental Processes and Ecological Remediation, Yantai Institute of Coastal Zone Research, Chinese Academy of Sciences, Yantai, 264003 China; 3 Key Laboratory of Cities’ Mitigation and Adaptation to Climate Change in Shanghai (CMA), College of Environmental Science and Engineering, Tongji University, Shanghai, 200092 China

## Abstract

To explore the utility of combining positive matrix factorization (PMF) with radiocarbon (^14^C) measurements for source apportionment, we applied PM_2.5_ data collected for 14 months at a national background station in North China to PMF models. The solutions were compared to ^14^C results of four seasonally averaged samples and three outlier samples. Comparing the most readily interpretable PMF solutions and ^14^C results revealed that PMF modeling was well able to capture the source patterns of PM_2.5_ with two and three irrelevant source classifications for the seasonal and outlier samples. The contribution of sources that could not be classified as either fossil or non-fossil sources in the PMF solution, and the errors between the modeled and measured concentrations weakened the effectiveness of the comparison. Based on these two factors, we developed an index for selecting the most suitable ^14^C measurement samples for combining with the PMF model. Then we examined the potential for coupling PMF modeling and ^14^C data with a constrained PMF run using the ^14^C data as a priori information. The restricted run could provide a more reliable solution; however, the PMF model must provide a flexible dialog to input the priori restrictions for executing the constraint simulation.

## Introduction

Atmospheric fine particles with aerodynamic diameters <2.5 μm (PM_2.5_) are recognized as key pollutants associated with increased rates of mortality and morbidity for respiratory and cardiovascular diseases^1^. Reliable source apportionment of PM_2.5_ is vital to design effective programs and strategies to reduce PM_2.5_ concentrations in ambient air. However, source apportionment is complicated, because source identification and quantification generally cannot be directly measured or monitored^2^. These limitations have encouraged the development of novel approaches for the source apportionment of PM_2.5_^3^.

Receptor modeling is one method used for the source apportionment of PM_2.5_^4^. Widely used receptor models include principal component analysis/multiple linear regression, unmix, positive matrix factorization (PMF), and chemical mass balance^3–5^. These models apportion PM_2.5_ to sources by decomposing a matrix of speciated PM_2.5_ sample data collected from one or more receptor sites. In general, these models provide interpretable results, but the reliability of the results is not guaranteed^3^. To enhance the confidence level of the source apportionments or evaluate model uncertainties, receptor models can be combined with other methods. There are two main types of combinations. The first type uses a dataset of PM_2.5_ speciation measurements to drive different receptor models (or other types of models) for the source apportionment. For example, Song *et al*. used two receptor models and Bove *et al*. used one receptor model and a chemical transport model to apportion PM_2.5_ to sources^6, 7^. Balachandran *et al*. developed specific source profiles using Bayesian-based ensemble averaging of source impacts from three receptor models and one chemical transport model to apportion PM_2.5_ to sources^2^. The second type of combination employs a suite of different PM_2.5_ datasets to drive a receptor model. For example, Xie *et al*. used a PMF model with four different PM_2.5_ speciation datasets to evaluate the utility and consistency of the source apportionments^8^. Tao *et al*. loaded a single or combination of three biomass tracers with other species within PM_2.5_ into a PMF model to apportion PM_2.5_ to sources and to assess the uncertainties of the source contributions^9^. It should be noted that these combinations could be used to compare the coherence and uncertainty among different results, but cannot guarantee their reliability.

Carbon, nitrogen, and sulfur isotope measurements have been used to identify the sources of the respective PM_2.5_ components^10–12^, which could provide more reliable source signatures than the methods mentioned above. Radiocarbon (^14^C) measurements can be used to unambiguously quantify the contribution of fossil and non-fossil sources to carbonaceous components of PM_2.5_ because ^14^C becomes depleted in fossil sources due to their aging (half-life, 5,730 years), whereas non-fossil sources contain similar ^14^C levels as atmospheric carbon dioxide (CO_2_)^13–17^. However, there have been few attempts to combine ^14^C-derived source signatures with receptor models for the source apportionment of PM_2.5_^18, 19^.

We recently performed a preliminary exploration of combining PMF modeling and ^14^C measurements for the source apportionment of wintertime PM_2.5_^18^. To gain further insight into this method, we collected PM_2.5_ samples for 14 months at a national background atmospheric monitoring station on Tuoji Island, North China. Performing source apportionment at a background site could offer the opportunity to identify regional sources of PM_2.5_ in North China, where the adverse effects of PM_2.5_ on public health have triggered both public alarm and official concern^20^. Moreover, these sample data are appropriate for assessing PMF performance and the utility of combining PMF and ^14^C data to apportion PM_2.5_ to sources, because there are no obvious source emissions near the sampling site, and airborne pollutants are mixed and transformed well before reaching the sampling site. The main objectives of this study were: (1) to assess the capability of using PMF modeling for the source apportionment of PM_2.5_ by comparing PMF and ^14^C results; (2) to develop an index to select suitable ^14^C measurement samples for the most effective combination of ^14^C measurements with PMF to apportion PM_2.5_ to sources due to the high cost and complex pretreatment of ^14^C measurements; and (3) to propose additional improvements to PMF modeling software for coupling PMF and ^14^C data to increase the reliability of PM_2.5_ source apportionments.

## Results and Discussion

### General characteristics of PM_2.5_

During the entire sampling period, the mean ± standard deviation of the PM_2.5_ mass concentration was 57.7 ± 36.9 μg m^−3^ (range, 8.93–144 μg m^−3^). The dominant PM_2.5_ species were SO_4_^2−^, NO_3_^−^, OC, NH_4_^+^, EC, K^+^, and Fe, accounting for 17.3 ± 7.96%, 10.3 ± 6.48%, 7.98 ± 3.08%, 4.81 ± 2.27%, 4.12 ± 3.61%, 1.13 ± 0.617%, and 0.89 ± 0.64%, respectively, of the PM_2.5_ mass concentration. The seasonal averages of PM_2.5_ and most species concentrations were markedly higher in the spring of 2012 than the other four seasons (Supporting Information [SI] Text S1, Table S1, and Figures S1–S3).

### PMF modeling

In the BMRs with six to nine factors, all of the BS-DISP estimations showed that the largest decreases in Q(robust) were <1% of the Q(robust) of the corresponding BMR (SI Figure S4), suggesting that the results of the four BMRs could be considered the global optimum solutions for the corresponding number of factors. The F_peak_ model runs with strengths of −1.0, 1.0, and 1.5 showed increases in Q > 5% of Q(robust) from the four respective BMRs (SI Figure S5), indicating that these F_peak_ solutions should be removed from further consideration. However, the F_peak_ runs with strengths of −0.5 and 0.5 showed increases in Q < 5% of Q(robust) of the corresponding BMRs (SI Figure S5), suggesting that these F_peak_ solutions were acceptable for further analysis. Positive/negative F_peak_ strengths sharpened/smeared and smeared/sharpened the two solution matrices, respectively^21^. The comparable increases in Q values and opposite transformations of the two matrices suggested that there were no significant rotational effects of the two strengths of the BMR solutions. Among the four model experiments, the BMR with eight factors (BMR-8) showed the most significant declines in Q(true), Q(robust), and the ratio of Q(true)/Q(exp) (SI Figures S6 and S7). These declines suggested that the source apportionments of BMR-8 were more appropriate than those of BMR-6, BMR-7, and BMR-9.

The eight source factors in BMR-8 were identified based on the dominant species and prominent contributions of a source to each species on a specified sampling day. SI Text S2 and Figures S8–S14 elaborate on the recognition processes used in this study. The eight identified sources were traffic dust, shipping emissions, mineral dust, sea salt, vehicle emissions, industrial processes, coal combustion, and biomass burning. The total and seasonal contributions (%) of the eight sources to PM_2.5_ were calculated, and results are shown in SI Figure S15. Among these sources, biomass burning, shipping emissions, and coal combustion had the largest contributions, accounting for 27.5%, 17.5%, and 16.5%, respectively, of the PM_2.5_ mass concentration during the entire sampling period. These sources were followed by mineral dust (14.8%), vehicle emissions (10.4%), sea salt (4.8%), traffic dust (4.6%), and industrial processes (3.9%). The back trajectory, fire counts, and navigation activity information supported these identified sources. Details are presented in SI Text S3 and Figures S11–S19.

**Figure 1:**
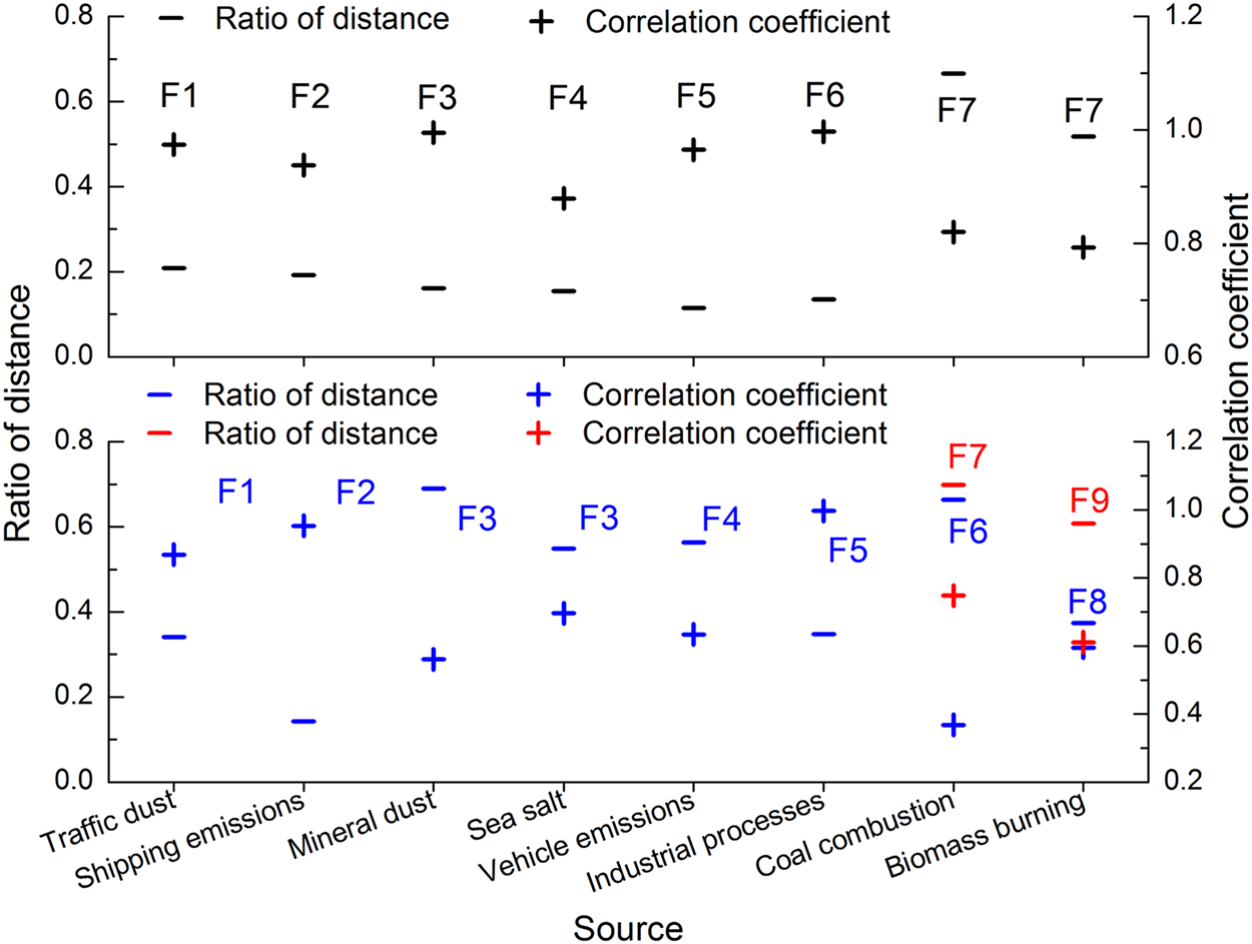
Ratios of the shortest distance between the standardized seven-factor (top panel) and nine-factor (bottom panel) source profiles from BMR-7 and BMR-9 and those from BMR-8 to the averages of their respective distance, and the Pearson correlation coefficients of the contribution time series related to the source profile with the shortest distance (labeled with the same color as that for the distance). For BMR-9, the source profile shows the shortest distance with two source profiles from BMR-8 (F3). Coal combustion and biomass burning identified in BMR-8 were each related to two different source profiles BMR-9.

To quickly identify the sources modeled by BMR-7 and BMR-9, the distances between their source profiles and that of BMR-8, and the Pearson correlations of their contribution time series with those of BMR-8 were calculated. Overall, most of the sources in BMR-8 matched the factors in BMR-7 and BMR-9 well. Six sources in BMR-7 were closely related to six sources identified by BMR-8, while one factor merged the two remaining sources (coal combustion and biomass burning) in BMR-8, characterized by their shortest distances and highest correlation coefficients (Fig. 1, detailed data are listed in SI Tables S2 and S3). This combined source had a lower contribution fraction to PM_2.5_ than the sum of the two separate sources (coal combustion and biomass burning) in BMR-8 (SI Figure S18). Among the eight sources identified by BMR-8, traffic dust, shipping emissions, vehicle emissions, and industrial processes matched the factors in BMR-9 well, characterized by their shortest distances and high correlation coefficients (Fig. 1, detailed data are listed in SI Tables S4 and S5). Both mineral dust and sea salt had the shortest distances and highest correlation coefficients with one factor in BMR-9, indicating that these two sources were combined in BMR-9 (Fig. 1). Also, coal combustion and biomass burning identified by BMR-8 each matched two separate factors in BMR-9. The two factors in BMR-9 related to coal combustion were industrial coal and domestic coal because their contribution time series correlated more with those of industrial processes and biomass burning, respectively (SI Table S5). Similarly, the two factors in BMR-9 associated with biomass burning were domestic biofuel and open biomass burning (SI Table S5). This source separation resulted in significant increases in the contribution of biomass burning and marked decreases in the contribution of coal combustion (SI Figure S19). We did not use the differences in the source contributions modeled by the three BMRs to determine the best solution among the three models. In BMR-7, coal combustion and biomass burning were inappropriately combined as a hybrid source, because they represent different types of emission sources and different primary contributors of PM_2.5_, suggesting that BMR-7 did not provide the optimal solution. This was supported by the initial mathematical analysis, which showed that BMR-8 provided the most physically interpretable results.

### PMF performance assessment

**Figure 2:**
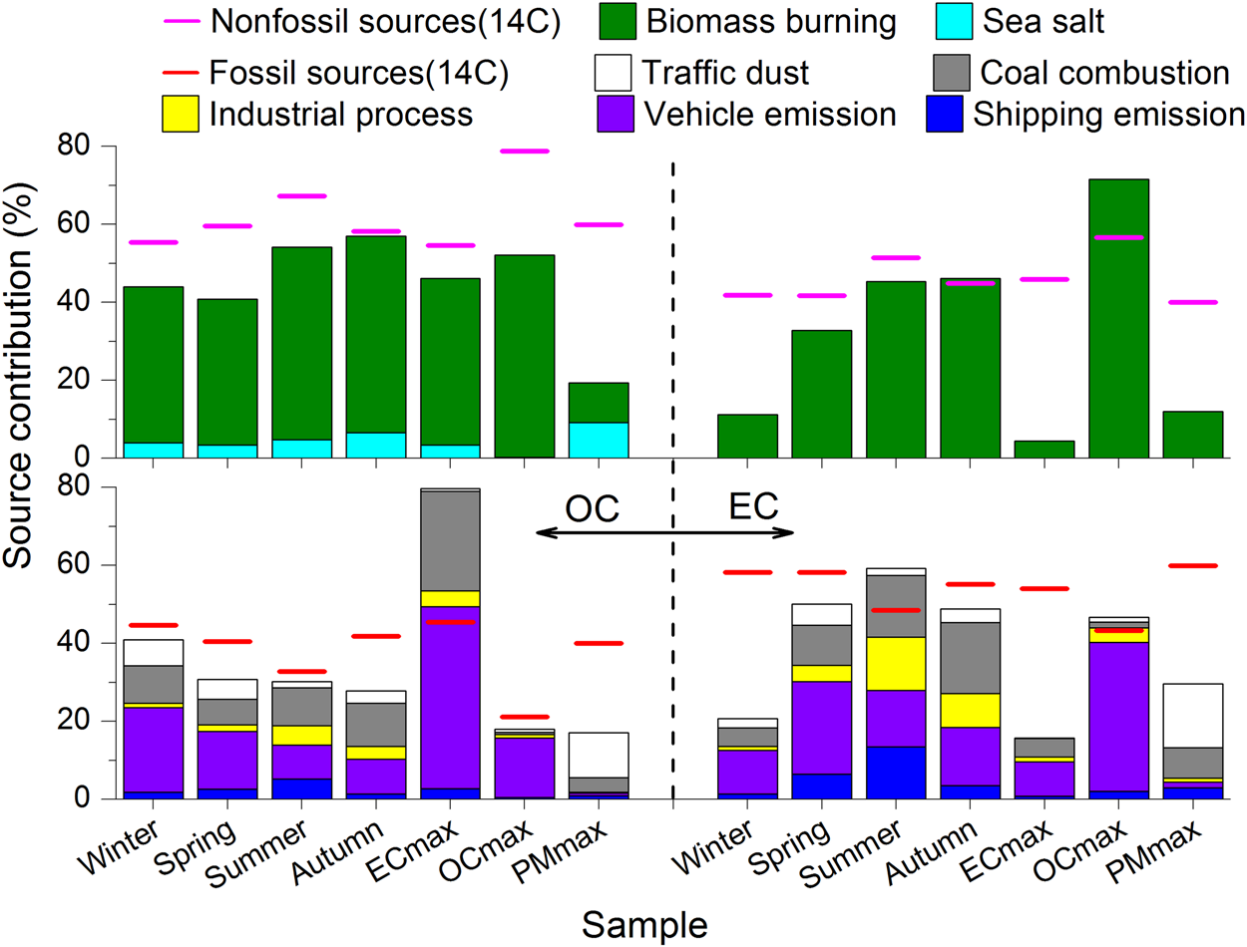
Comparison of the source apportionment of OC and EC classified from the PMF results and determined from the ^14^C measurements. Biomass burning and sea salt identified by PMF were combined as a non-fossil source, while coal combustion, industrial processes, vehicle emissions, and shipping emissions were merged as fossil sources for the comparison. Mineral dust was not included because it was considered a hybrid of non-fossil and fossil sources, and traffic dust was included as an additional fossil source in the comparison.

The model capacity was assessed by comparing the source contributions modeled by BMR-8 and BMR-9 with the ^14^C results. We did not consider BMR-7 for this evaluation, because the model included a source that combined fossil carbon (coal combustion) and non-fossil carbon (biomass burning) sources. According to the source types identified by BMR-8, coal combustion, industrial processes, vehicle emissions, and shipping emissions were ranked as fossil carbon sources, while sea salt as a marine biogenic source was merged with biomass burning as a non-fossil source. Mineral dust and traffic dust were not considered in this classification, because they could not be apportioned quantitatively into fossil and non-fossil sources. Figure 2 shows the contributions of the non-fossil and fossil sources categorized from the BMR-8 results and the corresponding ^14^C results for the seven samples. The source contribution from the BMR-8 results was calculated based on Equation (7) in the section of methods. For the four seasonal samples, from the winter of 2011 to autumn of 2012, the results of the non-fossil sources classified from BMR-8 accounted for 44.0%, 40.9%, 54.2%, and 57.0% of OC concentrations and 11.3%, 32.8%, 45.3%, and 46.2% of EC concentrations, respectively. Correspondingly, the categorized BMR-8 results showed that 34.3%, 25.6%, 28.6%, and 24.7% of OC and 18.4%, 44.7%, 57.4%, and 45.4% of EC were attributed to fossil sources, respectively. Most of the source contributions were lower than the corresponding ^14^C fractions. The underestimations were not considered to indicate inadequate capacity of the PMF model, because mineral dust and traffic dust were not taken into account for the comparison. In contrast, the overestimation of the source contributions classified from the PMF results could be considered irrelevant source apportionments of the PMF model. There were two source contribution overestimations of 8.9% and 1.3% for a fossil source of EC in summer and a non-fossil source of EC in autumn, respectively. This indicated that non-fossil sources were irrelevantly classified as fossil sources in the former case and fossil sources were categorized inappropriately as non-fossil sources in the latter. Traffic dust is thought to contain a higher fraction of fossil carbon than mineral dust^22^. Therefore, to further estimate PMF performance, we classified traffic dust as an additional fossil carbon source and re-compared the source contributions. The contribution of vehicle dust was added to the stack columns in Fig. 2. After this addition, the gaps in the source contributions of BMR-8 and ^14^C results decreased significantly, while the irrelevant source classifications increased non-significantly, indicating that the PMF model captured the primary source patterns of PM_2.5_ well. SI Figure S20 displays the comparison of the^14^C and PMF results calculated based on Equation (6) in the section of methods. The comparison shows a similar pattern as that in Fig. 2. This similarity indicated that the PMF model reproduced the seasonally measured concentrations well, and the differences in the source contributions modeled by BMR-8 and the ^14^C results were attributed to the effects of mineral dust rather than errors between the modeled and measured concentrations.

A similar comparison to that described above was performed for OC and EC in the outlier samples. Regardless of whether traffic dust was considered a fossil source, only three overestimations were observed, among which the two most marked overestimations were the contribution of fossil sources to OC (33.7% and 34.3% without and with consideration of vehicle dust as a fossil source) in the sample with the highest EC concentration and the contribution of non-fossil sources to EC (14.8%) in the sample with the highest OC concentration. In addition, a slight overestimation was observed for the contribution of fossil fuel combustion to EC (2.2% and 3.4% without and with consideration of vehicle dust as a fossil carbon source) in the sample with the highest OC concentration (Fig. 2). The comparison based on Equations (6) and (7) showed that the differences in source contributions for the samples with the highest OC and EC concentrations were the result of the contributions of both mineral dust and model error, while the difference in the sample with the highest PM_2.5_ concentration was mainly driven by mineral dust.

We compared the source contributions modeled by BMR-9 with the ^14^C results. For this comparison, domestic coal, industrial coal, industrial processes, vehicle emissions, and shipping emissions identified from BMR-9 were ranked as fossil sources, whereas domestic biomass burning and open biomass burning were classified as non-fossil sources. Traffic dust and the hybrid mineral dust–sea salt source were not considered. There were more overestimations in this comparison than that of BMR-8 (SI Figure S21), suggesting that the BMR-8 model provided a more reliable source apportionment than BMR-9.

### Index development

We calculated the contribution fractions of non-fossil and fossil sources classified from the BMRs based on the corresponding measured concentrations, as shown in Equation (7) in the section of methods. The measured concentration can be replaced by the corresponding modeled concentration, and Equation (7) can be rewritten as:

1 $${R}_{ij}=\sum _{k=1}^{{\rm{n}}}{g}_{ik}{f}_{kj}/(\sum _{k=1}^{p}{g}_{ik}{f}_{kj}+{e}_{ij}),$$

where *e* is the error between the modeled and measured concentrations, and the other parameters are the same as those in Equation (6). According to the source classification of non-fossil and fossil sources, the sum of the two types of source contributions can be expressed as:

2 $${R}_{ij}=(Cn{f}_{ij}+Cf{f}_{ij})/(Cn{f}_{ij}+Cf{f}_{ij}+Cn{c}_{ij}+{e}_{ij}),$$

where *Cnf*, *Cff*, and *Cnc* are the concentrations of the non-fossil, fossil, and unclassified source contributions, respectively. *e* indicates the modeled error similar to that in Equation (1). Unlike the ^14^C results, the contribution fractions of non-fossil and fossil sources categorized from the PMF solution cannot add up to 1 because of the effects of unclassified sources and the error between the modeled and measured concentrations of OC and EC. The effects (*eff*) can be considered the fraction of the sum of concentrations from unclassified source contributions and the error between the modeled and measured concentrations as:

3 $$ef{f}_{ij}=(Cn{c}_{ij}+{e}_{ij})/(Cn{f}_{ij}+Cf{f}_{ij}+Cn{c}_{ij}+{e}_{ij})=(Cn{c}_{ij}+{e}_{ij})/{X}_{ij},$$

where the symbols have the same definitions as those in Equations (3) and (5).

Large *eff* values generally resulted in greater underestimations of the contributions of sources categorized from the PMF results. These underestimations cannot be directly used to assess PMF model performance, but suggest a reduction in the comparison effectiveness. ^14^C is usually measured for only a few independent samples due to the high cost and complicated pretreatment. This raises the question of how to select ^14^C measurement samples to yield a more valid assessment of model performance and better combination of the PMF model with ^14^C measurements. One feasible method is to develop an index to select the most efficient ^14^C measurement samples for the PMF assessment. Based on the analysis as mentioned earlier, we developed the following index, which is dimensionless:

4 $$Inde{x}_{j}=\sum _{i=1}^{n}(\frac{Cn{c}_{ij}+{e}_{ij}}{{X}_{ij}})\times \sum _{k=1}^{m}(\frac{Cn{c}_{ij}+{e}_{ij}}{{X}_{kj}}),$$

where the symbols have the same definitions as those in Equations (1), (2) and (7), and *n* and *m* represent the number of all species used for the PMF model and the number of carbonaceous species, respectively.

**Figure 3:**
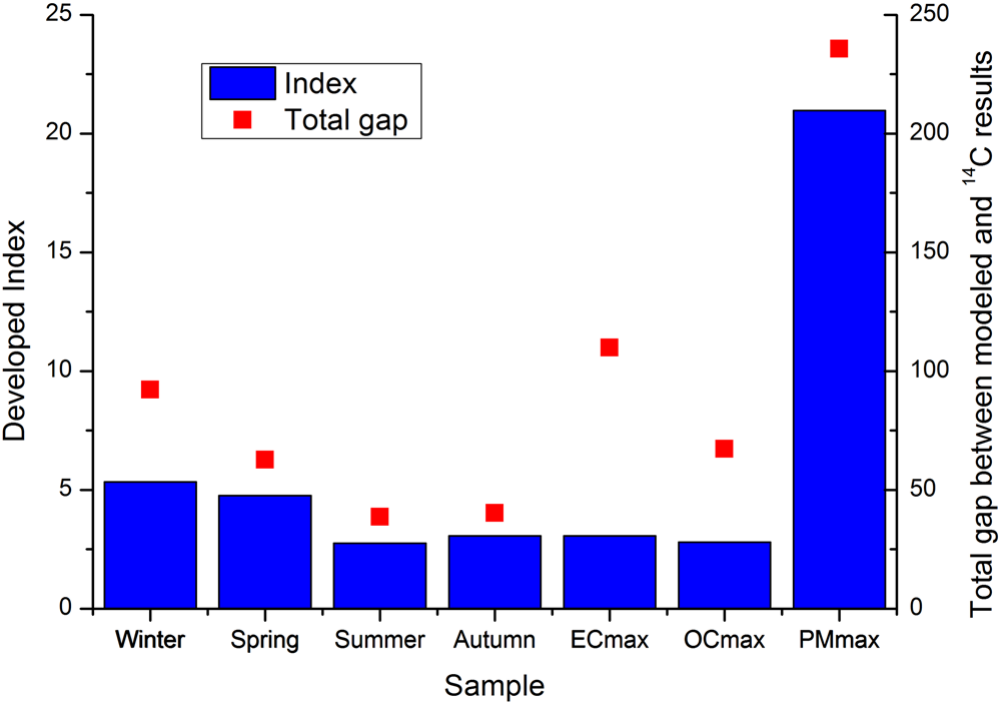
The developed index (dimensionless) used to select for ^14^C measurement and the total gap between the source contributions from the PMF and ^14^C results (%).

We calculated the index and total gaps in source contributions between the PMF and ^14^C results for each sample shown in Fig. 2, and results are presented in Fig. 3. Among the seven samples, that with the highest PM_2.5_ concentration had the largest total gap, which was attributed to the dominant contributions of mineral dust to PM_2.5_ (69% for OC and 66% for EC). Its irrelevance was characterized well by its high index value. The average summer sample had the smallest total gap, which was in agreement with its lowest index value, indicating that it was the best option among the seven samples. In addition, the index values of each sample were calculated using the data from BMR-7, BMR-8, and BMR-9. The variation in the indices presented similar trends as shown in SI Figure S22, indicating the stability of the developed index. This suggested that ^14^C measurement samples could be selected after several pre-simulations, rather than the final simulation, allowing for excellent performance of the assessment of the model’s capacity and the combination of the PMF model with ^14^C measurements. The stability of the index was attributed to similar trends in the errors between the modeled and measured PM_2.5_, OC, and EC concentrations and the modeled PM_2.5_ concentrations contributed by mineral dust and traffic dust among BMR-7, BMR-8, and BMR-9 (SI Figures S23–S25).

### Potential coupling between PMF and ^14^C measurement

The relationship between the PMF and ^14^C measurements discussed above was determined by a comparison, and suggested their independence. Further exploration focused on whether the ^14^C results could be coupled to the PMF as *a priori* information to provide more reliable model results using a constrained PMF model. According to the previous knowledge, the ^14^C results can be used to build constraint equations that specify a range of the total contribution of several sources (factors) to either OC or EC (species) in an individual or incorporative samples. For instance, the total contribution of coal combustion, industrial processes, vehicle emissions, and shipping emissions to OC in the sample with the highest EC concentration was less than 45.4%.

The EPA’s PMF 5.0 model uses the ME-2 program to identify the most optimal factor contributions and profiles. In ME-2, source contribution and composition knowledge as *a priori* information can be included as auxiliary terms of the object function to constrain a model run^21, 23^. Three types of constraints are included in the model. There are termed as Ratio, Mass Balance, and Custom. Users can select the Ratio option and use a constant ratio between two different species of a factor to constrain a model run. Users can build an equation by adding one or multiple factor-species on both sides of the equation to constrain a model run under the Mass Balance option. In the Custom option, users can specify a constraint by creating a customized equation to constrain a model run. The customized equation can be built based on either profile (with species as element) or contribution (with sample as element). All the three optional conversations cannot be used to build constraint equations based on the ^14^C results as mentioned above, indicating that the ^14^C results could not be coupled to EPA PMF 5.0 properly as *a priori* information to constrain a model run and to provide more reliable model results. There are two possibilities: the model software has not provided an appropriate conversation for coupling the ^14^C results, or the ME-2 algorithm cannot handle the constraint equations based on the ^14^C results. If the ME-2 algorithm can handle the constraint equations, it is vitality and recommended that the model software provides an appropriate conversation.

In order to assess the capacity of the ME-2 algorithm, we performed a preliminary constraint simulation based on BMR-8 to assess the variability of source contributions and availability of ^14^C results in the simulation. The preliminary constraint simulation used two additional constraint types (termed Pull Down Maximally and Pull Up Maximally) included in the model. Users can use the two options to pull down maximally and pull up maximally the contributions of one or multiple factors to all species for one or several samples. It should be noted that such a constrained modeling has incapable to provide a more reliable solution. Our concern was whether the range of source contributions from BMR-8 and the constrained modeling based on BMR-8 covers the ^14^C results. The coverage indicates that the ME-2 algorithm is able to handle the constraint modeling if the model software provides an appropriate conversation to build constraint equations.

As found above, the largest overestimations were observed in the samples with the highest EC and OC concentrations over the entire sampling period, and the largest overestimations of fossil sources to OC (33.7% and 34.3% without and with consideration of vehicle dust as a fossil source) were observed in the sample with the highest EC concentration. This sample had been collected on January 16, 2012. According to the overestimation, the contribution of two main fossil sources, coal combustion, and vehicle emission on the sampling day were pulled down maximally. Similarly, on June 6, 2012, when the OC concentration reached a maximum during the entire sampling period, biomass burning and sea salt were pulled up maximally. The results of the constrained run showed that Q(robust) and Q(true) increased by 0.51% and 0.63%, respectively, compared to those of BMR-8. Correspondingly, the source profiles and source contributions varied non-significantly compared to those in the base run (SI Figures S26 and S27).

**Figure 4:**
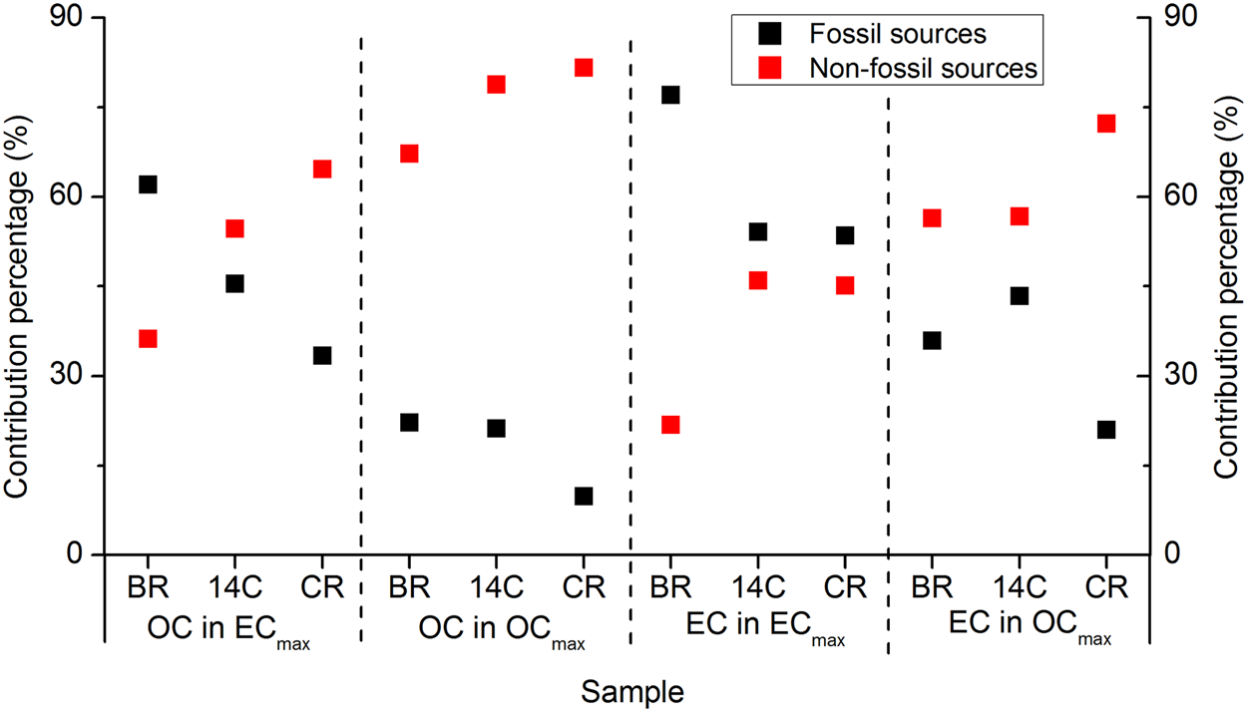
Contributions of fossil and non-fossil sources to OC and EC in the base model run (BR), constrained model run (CR), and ^14^C measurements (14 C).

The source contributions to OC and EC modeled by the BMR and the constrained model run were compared to the ^14^C results using the two samples with the highest OC and EC concentrations (Fig. 4). Most of the ^14^C results fell within the range between those modeled by the base and constrained model runs, suggesting that this model has the potential capacity to provide a reliable source assessment on PM_2.5_ in simulations when constrained by powerful *a priori* information, such as ^14^C data. However, to exploit this capability, the model must be able to provide more flexible conversations than those that currently exist. For instance, the sum contributions of several sources to one or several components measured in a sample or some samples collected for a period of time can be defined as a value or range. Once developed, this will provide a powerful tool for using PMF to apportion PM_2.5_ to sources, because knowledge on the sources of not only carbon-containing matter but also nitrogen- and sulfur-containing matter, in PM_2.5_ determined by their respective stable isotope measurements can be considered *a priori* information to constrain PMF models^10–12, 24, 25^. Nitrogen-, sulfur-, and carbon-containing matter account for the majority of PM_2.5_ mass concentrations^18, 26^. Therefore, if these three types of matter are handled as *a priori* information, the resulting constrained PMF models could provide highly reliable source apportionments of PM_2.5_, which is vital for designing PM_2.5_ control strategies.

## Conclusion

The utility of combining PMF with ^14^C for source apportionment was assessed using PM_2.5_ data collected for 14 months at a national atmospheric background station in North China. Four model experiments with six to nine factors were run and the most physically interpretable PMF solutions were determined by the comparison of their results. The best solutions were compared to the ^14^C results of four seasonally averaged samples and three outlier samples with the highest PM_2.5_, OC and EC concentrations. The comparison showed that PMF modeling can capture effectively the PM_2.5_ source patterns with two (8.9% and 1.3%) and three (33.7%, 14.8%, and 2.2%) inappropriate source apportionments for the seasonal and outlier samples. The contribution of sources that could not be classified as either fossil or non-fossil sources in the PMF solution and the errors between the modeled and measured concentrations weakened the validity of the comparison. Based on the two factors, an index was developed for choosing appropriate ^14^C measurement samples for coupling with PMF simulation. Potential coupling between PMF and ^14^C was examined by a constrained PMF run using ^14^C results as prior information. Results show that such a constrained run can obtain a more reliable solution, but PMF should provide a flexible dialog to execute the constraint. For instance, the sum contributions of several sources to one or several components measured in a sample or some samples collected for a period of time can be defined as a value or range. The design can be used to combine PMF models with prior information on nitrogen- and sulfur- containing material in PM_2.5_ determined by their respective stable isotope measurements, and exhibits the promising prospect for source apportionment.

## Methods

### Site description and sample collection

Tuoji Island is a small island with an area of 7.1 km^2^ located at the demarcation line between the Bohai Sea and the Yellow Sea. It is located 40 km north of the Shandong Peninsula, 300 km east of the Beijing-Tianjin-Hebei region, and 70 km south of the Liaodong Peninsula (SI, Figure S28). There is no industrial activity on the island, and the inhabitants support themselves by fishing. A national station for background atmospheric monitoring is located on the northwestern tip of Tuoji Island (38.188°N, 120.741°E) (SI Figure S29). The sampling platform at this station is about 10 m above ground level^27^.

At the sampling platform, PM_2.5_ samples were collected at the sampling platform every three days from December 2011 to January 2013. The sampling time started at 10:00 local standard time and sampling continued for 24 h. Samples were collected on quartz fiber filters (QM-A, 20.3 × 25.4 cm^2^, Whatman, heated at 500 °C for 8 h before use) using a high-volume sampler (Hi-Vol 3000; Ecotech, Australia) at a flow rate of 1.13 m^3^ min^−1^ ^27^. We selected samples from each month distributed evenly throughout the month and analyzed a total of 70 samples in this study.

### Chemical speciation and ^14^C measurements

The mass concentrations of PM_2.5_ were analyzed gravimetrically using a Sartorius MC5 electronic microbalance. Organic carbon (OC) and elemental carbon (EC) were analyzed with a Desert Research Institute Model 2001 carbon analyzer (Atmoslytic Inc., Calabasas, CA, USA). Water-soluble ions (i.e., sodium [Na^+^], ammonium [NH_4_^+^], potassium [K^+^], magnesium [Mg^2+^], calcium [Ca^2+^], chloride [Cl^−^], nitrate [NO_3_^−^], and sulfate [SO_4_^2−^]) were determined with ion chromatography (ICS-3000; Dionex Ltd., Sunnyvale, CA, USA). Metals (i.e., vanadium [V], manganese [Mn], iron [Fe], chromium [Cr], nickel [Ni], copper [Cu], zinc [Zn], arsenic [As], cadmium [Cd], and lead [Pb]) were measured with inductively coupled plasma mass spectrometry (ELAN DRC II; Perkin Elmer Ltd., Hong Kong). The methods have previously been described^28–30^ and details are provided in SI Text S4.

OC was split into the water-soluble organic carbon (WSOC) and water-insoluble organic carbon (WIOC) fractions. ^14^C was measured in WSOC, WIOC, and EC, respectively^17^. Briefly, WSOC and WIOC were separated with Milli-Q water, along with EC, and each fraction was subsequently converted into carbon dioxide (CO_2_). The CO_2_ from the three fractions was cryogenically trapped and reduced to graphite for accelerator mass spectrometry (AMS) target preparation^31–33^. The graphite targets were prepared using the graphitization line at the Guangzhou Institute of Geochemistry, Chinese Academy of Sciences, and the modern carbon fractions (ƒ_m_) in the graphite samples were determined with a compact accelerator mass spectrometry (AMS, National Electrostatics Corp., Middleton, WI, USA) at Peking University. To take the impact of the nuclear bomb in the 1950s and 1960s into account, the non-fossil carbon fraction (ƒ_c_) in the samples was defined as ƒ_c_ = ƒ_m_/1.10 for EC and ƒ_c_ = ƒ_m_/1.06 for OC and the fossil carbon fraction (ƒ_ƒ_) was defined as ƒ_ƒ_ = 1–ƒ_c_^34, 35^. This method was previously described in detail^18^ and is presented in SI Text S4.

### Source apportionment model and capacity assessment

We used the Environmental Protection Agency’s (EPA’s) PMF 5.0 model to explore combining PMF with ^14^C measurements for the source apportionment of PM_2.5_. The model decomposes a matrix into two matrices using multiple iterations of the Multilinear Engine (ME-2)^23^. The optimal solutions for a model run are identified as those with the smallest sums of the squared residuals between the products of the two matrices minus the original matrix divided by the corresponding uncertainty matrix. During the iteration, two random matrices are generated, and then modified systematically along the direction provided by the conjugate gradient algorithm to find the best-fitting solution^23^. The squared residuals are indicated as Q(true) and Q(robust) in the model. Q(true) is the goodness-of-fit parameter calculated from all of the points. Q(robust) is the goodness-of-fit parameter calculated after excluding points with an uncertainty-scaled residual >4 not fit by the model. The best-fitting solution from a set of random matrices is considered a local minimum, rather than the global minimum, for a decomposed matrix. To maximize the chance of reaching the global minimum, the model should be run many times with different starting points, and the solution with the smallest Q values among the runs is used as the optimal solution and is often called the base model run (BMR). The model’s guide suggests that the most optimal solution among 100 model runs can be considered the global minimum for the final source apportionment^36^. The ratio of Q(true)/Q(exp) is an effective parameter for assessing the PMF solution. In the model, the bootstrap enhanced by displacement of factor elements (BS-DISP) method is used to estimate the errors associated with both random and rotational ambiguity. If the change in Q of the BS-DISP estimation is <1% of the Q(robust) from the corresponding BMR, the BMR results are considered to have non-significant rotational ambiguity^36, 37^. F_peak_ can be used to assess whether the model results fill the solution space by rotating a given solution. If the increase in the Q value due to the F_peak_ rotation is >5% of the Q(robust) from the corresponding BMR, the BMR solution can be perceived as covering the full solution spaces^36, 37^. Details of these processes are described in the user guide of the model^31^.

In this study, 21 chemical species (OC, EC, NO_3_^−^, SO_4_^2−^, Cl^−^, NH_4_^+^, K^+^, Mg^2+^, Ca^2+^, Na^+^, As, Cr, Cd, Cu, Fe, Mn, Ni, Zn, Pb, V, and unrecognized components) were loaded into the model to quantitatively apportion PM_2.5_ to sources. The species method detection limits shown in SI Text S4 were used to calculate uncertainties for each sample according to the equation-based uncertainty method. Four model experiments with six to nine factors were each run 100 times. The best solution of each model experiment (i.e., the BMR) was identified as that with the minimum Q(robust) value. The errors associated with both random and rotational ambiguity of the four model experiments were examined from the respective BS-DISP estimations based on the respective BMRs. The solutions of the four BMRs were examined from the F_peak_ model run with strengths of −1.0, −0.5, 0.5, 1.0, and 1.5.

To combine PMF with ^14^C measurements for the source apportionment of PM_2.5_, we first focused on the PMF model performance by comparing fossil and non-fossil source contributions determined from the ^14^C analysis, classified as OC and EC from the PMF results using several specific samples. For the comparison, we used four samples merged seasonally and three outlier samples of ^14^C measurements. The seasonally merged samples were made by pooling sample punches of equal sizes cut from samples collected in the first four seasons of the study period. For the outlier samples, we selected the samples with the highest OC, EC, and PM_2.5_ concentrations in the entire sampling period. For the comparison, the ^14^C fraction of OC was calculated from the concentration-weighted WSOC and WIOC fractions as:

5 $${f}_{OC}=({f}_{WSOC}\times {C}_{WSOC}+{f}_{W{\rm{I}}OC}\times {C}_{WIOC})/({C}_{WSOC}+{C}_{WIOC}),$$

where ƒ_OC_, ƒ_WSOC_, and ƒ_WIOC_ are the non-fossil carbon fractions of OC, WSOC, and WIOC, respectively, and C_WSOC_ and C_WIOC_ are the WSOC and WIOC concentrations, respectively. The modeled source contributions were classified into two groups according to their fossil and non-fossil carbon sources. The contribution fractions of fossil or non-fossil carbon sources to OC and EC were subsequently compared to the ^14^C results of the seven samples. In our previous study^18^, the contribution fractions (R) of non-fossil or fossil sources to OC or EC classified from the PMF results were determined by:

6 $${R}_{ij}=\sum _{k=1}^{{\rm{n}}}{g}_{ik}{f}_{kj}/\sum _{k=1}^{p}{g}_{ik}{f}_{kj},$$

where *g* and *f* are the factor contributions and factor profiles, respectively, *i* represents the OC or EC species, *j* is a specified sample, *n* is the number of fossil or non-fossil carbon sources, and *p* is the number of all of the sources. The calculation is based on the total OC or EC concentrations based on the PMF model rather than the measured concentrations, indicating that the errors between the modeled and measured concentrations are ignored in these comparisons^18^. For a more reasonable comparison and assessment, in this study we replaced the modeled total concentration in Equation (6) with the corresponding measured concentration:

7 $${R}_{ij}=\sum _{k=1}^{{\rm{n}}}{g}_{ik}{f}_{kj}/{X}_{ij},$$

where *X*_*ij*_ is the measured OC or EC concentration and the other parameters are the same as those in Equation (6). Furthermore, we used backward trajectories (http://www.arl.noaa.gov/ready.html)^38^ and fire counts (https://firms.modaps.eosdis.nasa.gov/firemap/) to assess the potential sources of PM_2.5_. A distance analysis and Pearson correlation were used to quickly identify the source types and their contributions to PM_2.5_. Detailed methods are described in SI Text 5.

## Electronic supplementary material

Supplementary information
