# Supplementary material for: Combining Positive Matrix Factorization and Radiocarbon Measurements for Source Apportionment of PM2.5 from a National Background Site in North China
Source: Sci Rep. 2017 Sep 6;7:10648. doi: 10.1038/s41598-017-10762-8 (PMC5587569; doi:10.1038/s41598-017-10762-8)
Supplement: Supplementary file 1 — Supplementary information [file 41598_2017_10762_MOESM1_ESM.pdf]

Supporting Information (SI) for

Combining Positive Matrix Factorization and Radiocarbon Measurements for Source Apportionment of $PM_{2.5}$ from a National Background Site in North China

Xiaoping Wang[1,2], Zheng Zong[2], Chongguo Tian[2*], Yingjun Chen[3], Chunling Luo[1], Jun Li[1], Gan Zhang[1], Yongming Luo[2]

[1] State Key Laboratory of Organic Geochemistry, Guangzhou Institute of Geochemistry, Chinese Academy of Sciences, Guangzhou, 510640, China

[2] Key Laboratory of Coastal Zone Environmental Processes and Ecological Remediation, Yantai Institute of Coastal Zone Research, Chinese Academy of Sciences, Yantai, 264003, China

[3] Key Laboratory of Cities' Mitigation and Adaptation to Climate Change in Shanghai (CMA), College of Environmental Science and Engineering, Tongji University, Shanghai, 200092, China

* Corresponding author:

Chongguo Tian, Yantai Institute of Coastal Zone Research, CAS. Phone: +86-535-2109-160; Fax: +86-535-2109-000; e-mail: cgtian@yic.ac.cn

Number of Pages: 38

Number of Texts: 5

Number of Tables: 5

Number of Figures: 29

Text S1 Concentration and Seasonal Variation

Averaged Concentrations. Table S1 lists average and seasonal concentrations of analyzed chemical components in $PM_{2.5}$ collected at the Tuoji Island during the sampling period. The $PM_{2.5}$ mass concentrations ranged from 8.93 to 144 μg m^{-3} with an average of 57.7 ± 36.9 μg m^{-3}. A total of 65% and 30% of the measured $PM_{2.5}$ levels exceeded the First Grade National Standard (35 μg m^{-3}, 24h) and Second Grade National Standard (75 μg m^{-3}, 24h) of China, respectively, even though the samples were collected at a national station for background atmospheric monitoring. The dominant species of $PM_{2.5}$ were SO_4^{2-} (9.66 ± 7.93 μg m^{-3}), NO_3^- (7.07 ± 7.17 μg m^{-3}), OC (4.38 ± 3.14 μg C m^{-3}), NH_4^+ (2.63 ± 1.94 μg m^{-3}), EC (2.21 ± 2.32 μg C m^{-3}), K^+ (0.691 ± 0.683 μg m^{-3}), and Fe (0.527 ± 0.545 μg m^{-3}), accounting for 17.3 ± 7.96%, 10.3 ± 6.48%, 7.98 ± 3.08%, 4.81 ± 2.27%, 4.12 ± 3.61%, 1.13 ± 0.617%, and 0.888 ± 0.639% of total $PM_{2.5}$, respectively.

Seasonal Variation. Seasonal trend shows that the highest $PM_{2.5}$ concentration (86.0 ± 38.0 μg m^{-3}) occurred in the spring of 2012, followed by the winter of 2012 (57.0 ± 38.9 μg m^{-3}), the summer of 2012 (52.4 ± 35.2 μg m^{-3}), the autumn of 2012 (49.2 ± 33.8 μg m^{-3}), and the winter of 2011 (46.1 ± 30.2 μg m^{-3}), respectively (Table S1). The concentrations of dominant chemical components in $PM_{2.5}$ showed similar trend with $PM_{2.5}$, such as the highest concentrations of SO_4^{2-} (12.1 ± 9.65 μg m^{-3}), NO_3^- (10.9 ± 8.07 μg m^{-3}), K^+ (0.802 ± 0.431 μg m^{-3}), and Fe (0.993 ± 0.691 μg m^{-3}), and the second highest concentrations of OC (5.45 ± 2.19 μg C m^{-3}), EC (1.99 ± 0.99 μg C m^{-3}), and NH_4^+ (3.13 ± 2.16 μg m^{-3}), in the spring of 2012.

Source Signature from Several Tracers. Variation in concentrations of several tracer components in $PM_{2.5}$ can reflect the change of source signals. K^+ is always regarded as a signature of biomass burning[1]. Seasonal K^+ concentrations gradually decreased from spring to winter in 2012, (0.802 ± 0.431 μg m^{-3}, 0.798 ± 1.23 μg m^{-3}, 0.691 ± 0.522 μg m^{-3}, 0.614 ± 0.357 μg m^{-3}), followed by the winter of 2011 (0.554 ± 0.470 μg m^{-3}). The descending sequence indicates that the strongest signature of biomass

burning occurred in the spring, and the signature receded continually up to winter. Ca^{2+} is often considered as a signature of mineral dust. Seasonal variations in Ca^{2+} concentrations listed in Table S1 indicate that the strongest impact of mineral dust on $PM_{2.5}$ at Tuoji Island occurred in the spring, followed by the autumn, the winter, and the summer. Previous study also reported that Asian dust events primarily occur in springtime and the dust storms can arrive at downwind areas, including Bohai sea[2]. Occurrence and transport of dust particles are closely related to weather conditions, especially wind speeds[3]. High wind speeds in spring and autumn (Figure S3) provided a favorable transport of dust aerosols to the island from origins. Na^{+} is regarded as a signature of sea salt near the seaside. The contribution of sea salt to atmospheric particles is also inextricably linked with high wind speed. Seasonal Na^{+} concentrations decreased in the descending sequence from autumn, winter, spring in 2012, winter in 2011, and summer in 2012, agreeing well with wind speed at the island as shown in Figure S3. Sea salt emissions are comprised of not only Na^{+}, but also SO_4^{2-}, K^{+}, and Ca^{2+}. The amounts of different chemical species in sea salt emissions are often determined by using Na^{+} as the tracer of sea salt; the amounts of these species from non-sea salt (nss-) emissions can be expressed as:

$$nss\text{-}x = x - [Na^{+}] \times a \qquad (1)$$

where x indicates the SO_4^{2-}, K^{+}, and Ca^{2+} concentrations, and a is the typical equivalent concentration ratio of the corresponding species to Na^{+} in average seawater: SO_4^{2-}/Na^{+} (0.250), K^{+}/Na^{+} (0.036), and Ca^{2+}/Na^{+} (0.038)[4]. If calculated concentration of non-sea salt chemical species is negative, then no excess species exist. According to the calculation, non-sea salt sources of the three chemical species in the five seasons contributed over 90% for corresponding total concentrations, as shown in Figure S1-3. The assessment indicates that minor contribution of sea salt to $PM_{2.5}$ and possibly intensive impact of anthropogenic emission on atmospheric particles at the island. From the winter of 2011 to the winter of 2012, the percentages of nss-K^{+} to $PM_{2.5}$ were 1.09 ± 0.46%, 0.92 ± 0.44%, 1.05 ± 1.03%, 1.25 ± 0.51%, and 1.15 ±

0.47%, respectively. The percentages indicate a relative higher contribution of biomass burning to $PM_{2.5}$ concentration in the autumn than other four seasons. The percentages of nss-Ca to $PM_{2.5}$ were 0.18 ± 0.39%, 0.62 ± 0.73%, 0.18 ± 0.16%, 0.31 ± 0.54%, and 0.12 ± 0.13%, respectively, suggesting the highest contribution of mineral dust to $PM_{2.5}$ in the spring.

Table S1. $PM_{2.5}$ chemical components at Tuoji Island during the sampling period (μg m^{-3})

Species	Winter,2011	Spring, 2012	Summer, 2012	Autumn, 2012	Winter, 2012	Average
$PM_{2.5}$	46.1	86.0	52.4	49.2	57.0	57.6
OC	3.69	5.45	3.30	4.06	5.98	4.38
EC	4.19	1.99	1.15	1.42	1.88	2.21
SO_4^{2-}	7.13	12.1	10.5	9.37	11.4	9.92
NO_3^-	7.16	10.9	3.73	7.19	6.48	7.07
NH_4^+	2.49	3.13	2.24	1.99	3.56	2.63
K^+	0.554	0.802	0.798	0.691	0.614	0.691
Cl^-	0.745	0.778	0.220	0.324	0.294	0.488
Na^+	0.353	0.425	0.314	0.531	0.512	0.419
Ca^{2+}	0.191	0.633	0.187	0.331	0.127	0.295
Mg^{2+}	0.051	0.066	0.014	0.034	0.054	0.043
Fe	0.443	0.993	0.331	0.498	0.366	0.527
Mn	0.126	0.268	0.242	0.272	0.065	0.198
Zn	0.076	0.116	0.121	0.111	0.095	0.103
Pb	0.082	0.087	0.115	0.102	0.105	0.098
Cu	0.008	0.013	0.018	0.011	0.009	0.012
V	0.004	0.008	0.008	0.005	0.003	0.006
As	0.004	0.005	0.005	0.004	0.006	0.005
Ni	0.003	0.006	0.005	0.004	0.004	0.004
Cr	0.003	0.005	0.004	0.004	0.004	0.004
Cd	0.001	0.001	0.001	0.001	0.001	0.001

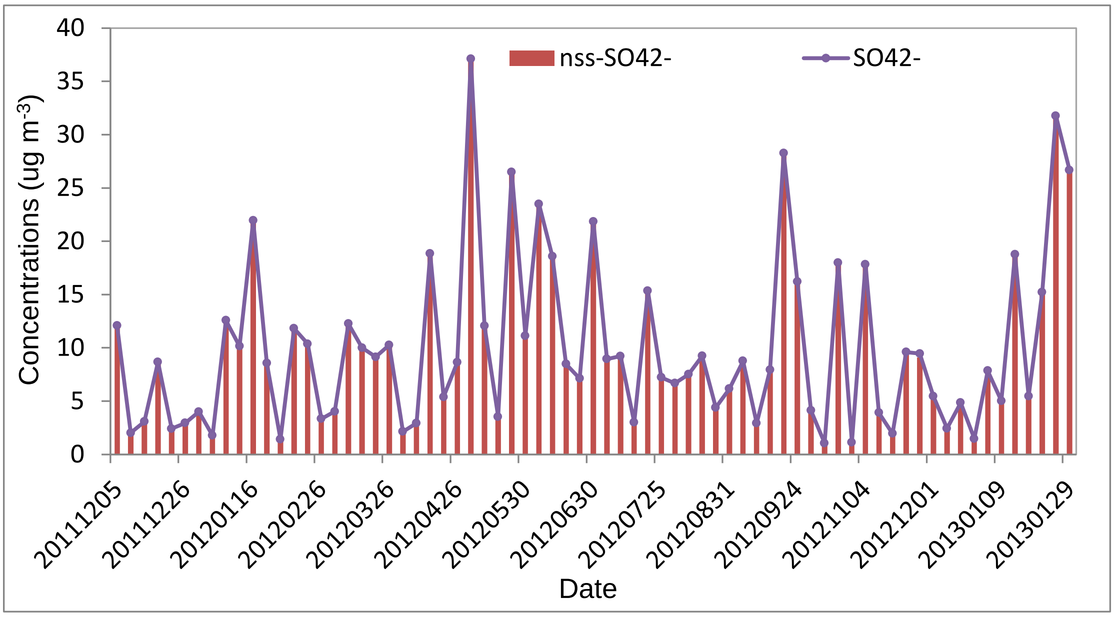

Figure S1. Total SO_4^{2-} concentration and the concentration deducted the parts from sea salt (nss-SO_4^{2-}) at Tuoji Island during the sampling period

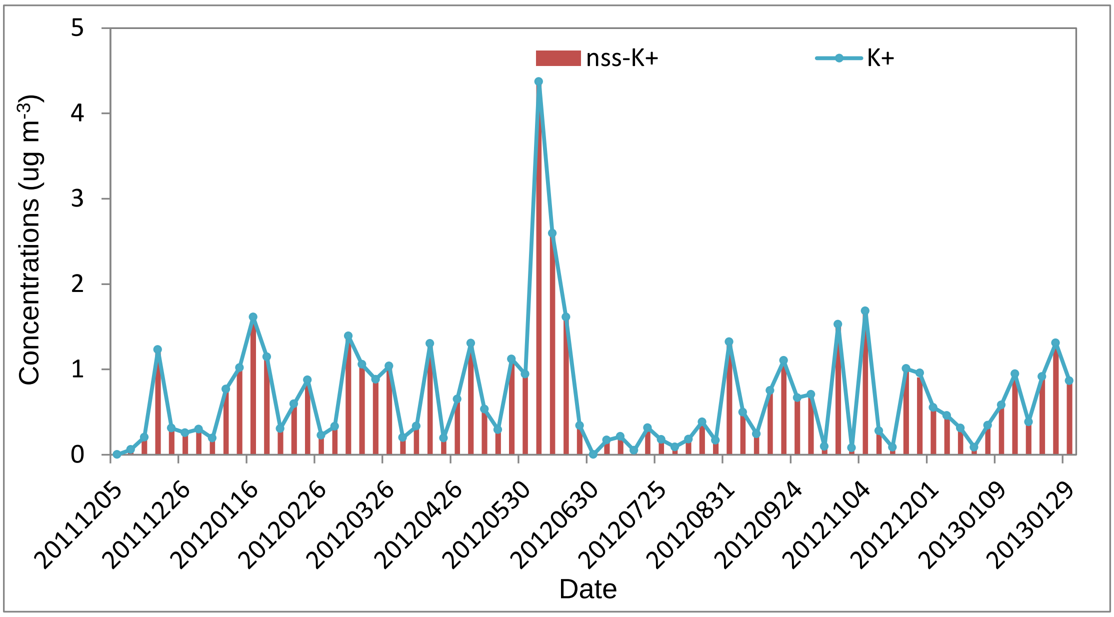

Figure S2. Total K^+ concentration and the concentration deducted the parts from sea salt (nss-K^+) at Tuoji Island during the sampling period

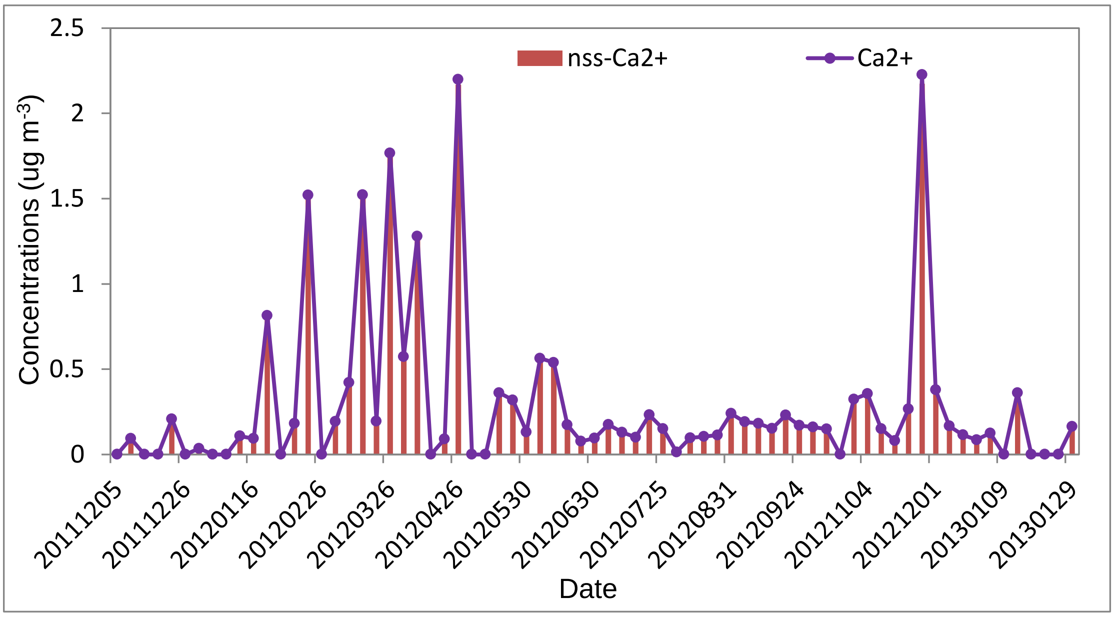

Figure S3. Total Ca^{2+} concentration and the concentration deducted the parts from sea salt (nss-Ca^{2+}) at Tuoji Island during the sampling period

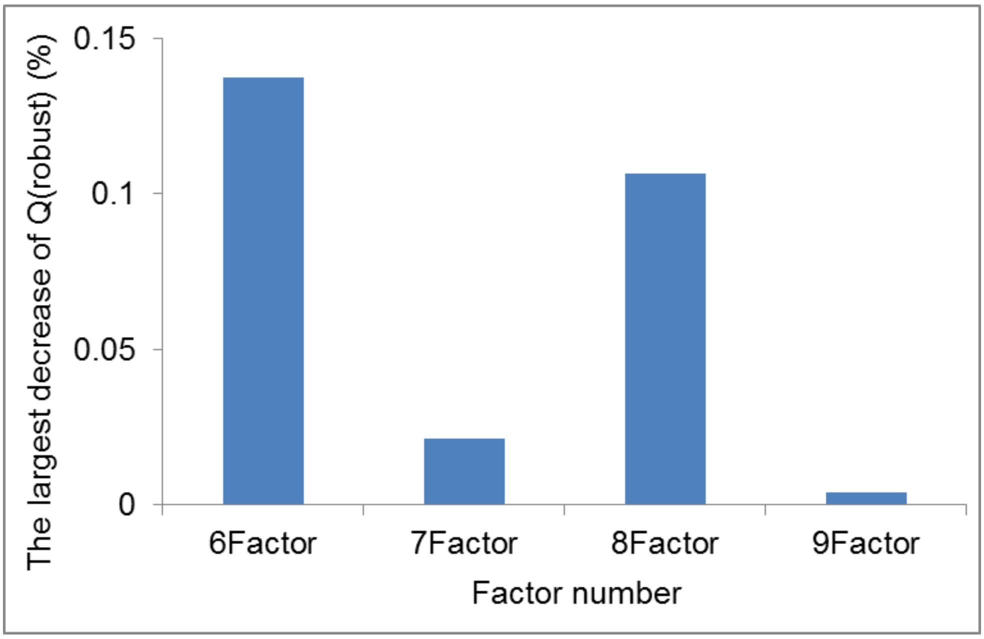

Figure S4. The largest decease of Q(robust) values calculated by the BS-DISP estimation based on the four base model runs with factors from six to nine

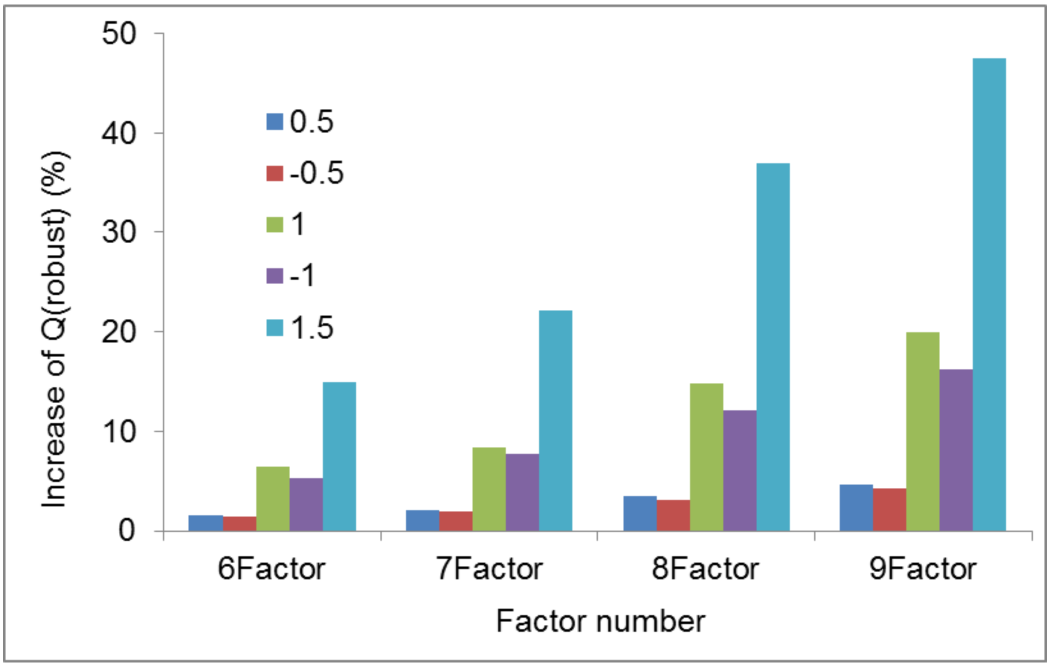

Figure S5. The largest decease of Q(robust) calculated by the F_{peak} estimation based on the four base model runs with factors from six to nine

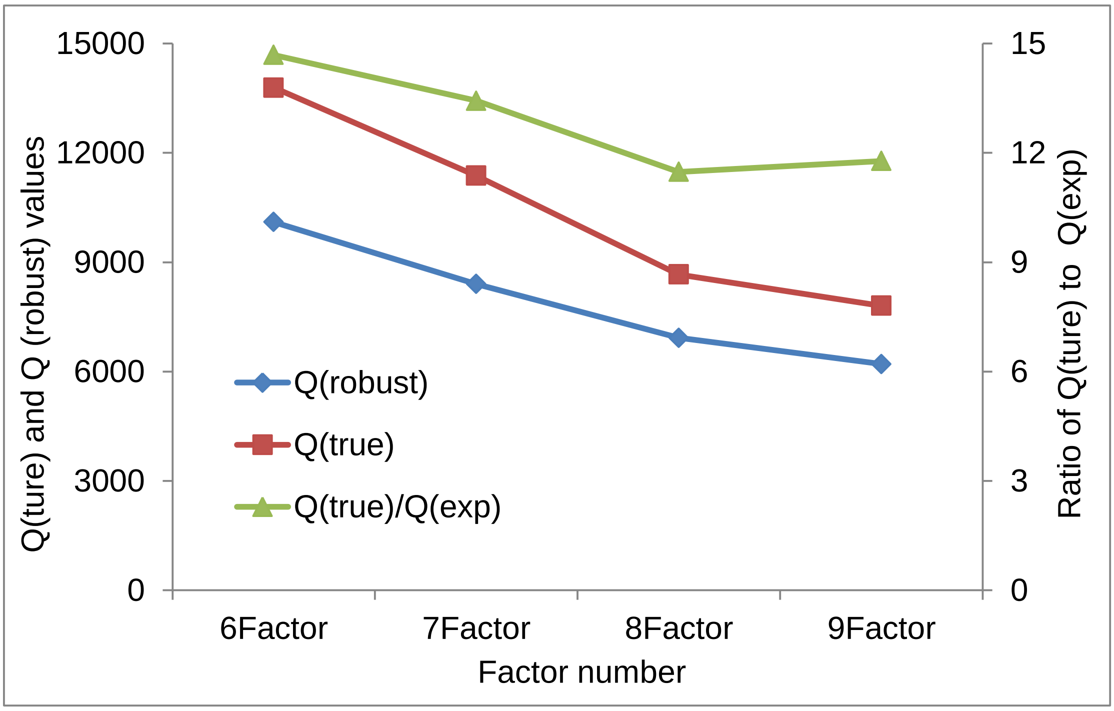

Figure S6. The changes of Q values from the four base model runs with factors from six to nine

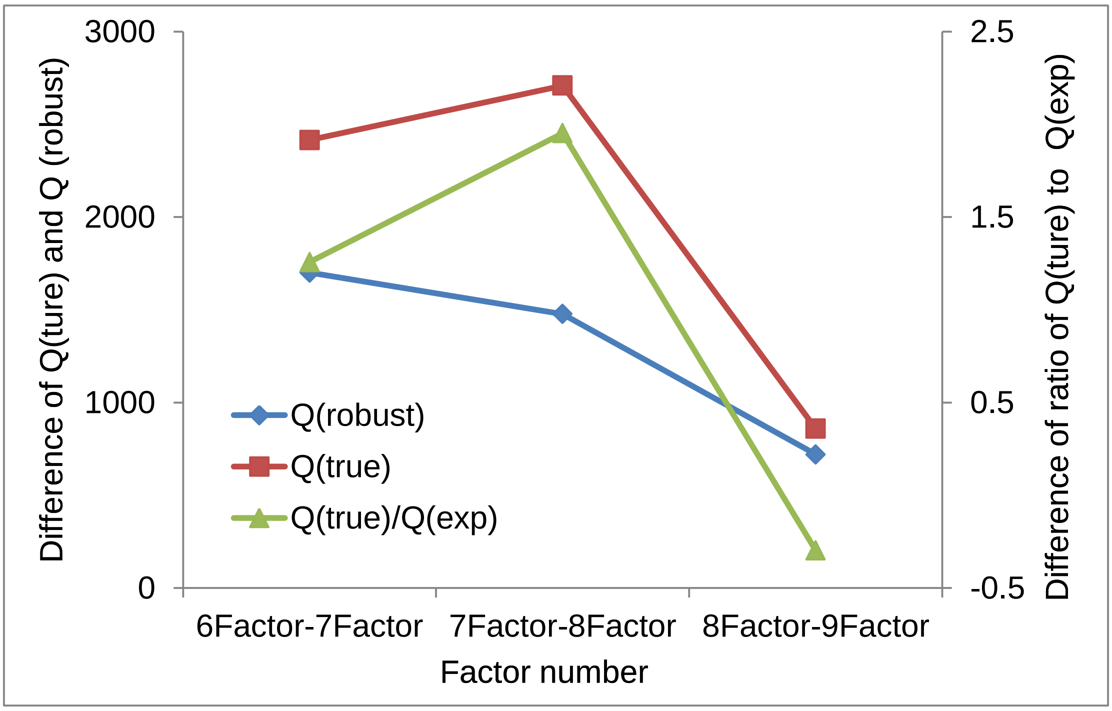

Figure S7. The difference of Q values between two adjacent factors from the four base model runs with factors from six to nine

Text S2 Source Apportionment of $PM_{2.5}$ by PMF

Figure S8 shows concentration profile and source contribution modeled by PMF base model run with eight factors. **Factor 1** was characterized by high Mg^{2+}, Fe and Zn. Vehicle dust is considered as an important source of atmospheric Zn pollution because it can be emitted from tire and brake abrasion, wearing and corrosion from anticorrosion galvanized automobile sheet, and re-entrainment dust enriched with Zn, showing an anthropogenic source pattern[5]. These chemicals of Mg^{2+} and Fe are typical crustal elements, showing mainly a natural source pattern[6]. Thus, the source factor shows a mixed pattern of natural and anthropogenic emissions and was identified as traffic dust under the relatively high contribution of vehicle emission to $PM_{2.5}$ concentration in the present study.

Factor 2 was characterized by high loadings of V and Ni, and a high V/Ni ratio. A high loading of these two metals is typically associated with emissions from residual oil, probably derived from shipping activities and some industrial processes[7,

[8]. In addition, a V/Ni ratio of more than 0.7 is always considered a signature of $PM_{2.5}$ influenced by shipping emissions[9]. The ratio of measured V concentration to measured Ni concentration ranged from 0.2 to 2.51, with an average of 1.42 and a standard deviation of 0.42 during the entire sampling period, indicating an obvious contribution of shipping emissions to $PM_{2.5}$ at the island. The average ratio of V/Ni calculated from the PMF source profile was 1.02, which was the highest value among those derived from the eight sources. Therefore, the source factor was treated as shipping emissions.

Factor 3 was characterized by high proportion of Ca^{2+} and relatively high proportion of Mn and Fe. These chemicals are typical crustal elements and often used as markers of soil dust[6]. The high loadings of the source contribution occurred in spring, 2012, as shown in Figure S9. The time period is consistent with the period with the high frequency of Asian dust events [2]. Furthermore, a high loading of source contribution from this factor was found on November 27, 2012 (Figure S9). To explore the source type, 72-h back trajectories for this day are presented in Figure S10. The air mass was found from the northwest region of China and the transport distance about 3200 km in 72 h, which is commensurate with an average wind speed of 12 m s^{-1}. The average of daily wind speed (see SI Figure S11) and the highest wind speed in 10 minutes observed at the sampling platform of the national station for background atmospheric monitoring were 9.0 and 14.2 m s^{-1}, respectively. High wind speed is favorable to diffuse atmospheric pollutants from anthropogenic sources and increases likely the contribution of nature emissions, such as dust and sea salt[3].

Factor 4 was characterized by high loadings of Na^{+} and Cl^{-}. The two components are treated as makers of sea salt aerosol (Figure S8). The highest contribution occurred on November 27, 2012, which was similar to the high loading of mineral dust as shown in Figure S9. The loading contribution was related to sea-salt aerosol emission produced by mechanical disruption of the ocean surface under high wind speed on the day[10]. In addition, the concentration ratios of Cl^{-}/Na^{+}

and SO_4^{2+}/Na^+ calculated from the PMF source profile were 1.71 and 0.23, respectively, similar to the corresponding ratios of the species (1.80 and 0.25, respectively) in average seawater. The source contributed OC concentrations but provided no EC contribution. This indicates the source consists of sea-spray organic aerosol, which came from the marine biogenic activities[11].

Factor 5 was characterized by high loadings of NO_3^-, EC, NH_4^+, and OC, which matched a vehicle emission profile[6]. Generally, NO_3^-, SO_4^{2-}, OC and EC are mainly from engine exhaust emissions, and NH_4^+ is from vehicles equipped with three-way catalytic converters[12]. Not only Zn and Cu, but also Pb and Cd are emitted directly bounded particles from exhaust[13]. In addition, the high NO_3^-/SO_4^{2-} ratio of 3.73 calculated by the PMF result suggested high temperature burning and vehicle emissions. In fact traffic emission has attracted considerable concern in the megacities of China (e.g., Beijing and Shanghai) due to the remarkable growth of vehicle numbers in China[14-16]. In Beijing in 2012, on-road vehicles were estimated to be the largest local emission source and contributed 22% of $PM_{2.5}$, including primary and secondary fine particles but vehicle-induced road dust[15]. This source was the largest contributor of NO_3^-, which contributed 48% during the sampling period. The contribution was higher than 25% of NO_x emitted by traffic sectors in North China in 2003, an expected increase of the contribution due to the rapid rise of vehicles in North China in recent years[17]. This factor was the prevalent anthropogenic $PM_{2.5}$ source in North China, with an average contribution of 10% during the sampling period. The contribution was lower than that in Beijing[15], agreeing with the regional contribution characteristic in our study, rather than ones in large cities. Moreover, vehicle equipped with three-way catalytic converters could be a more and more important source for NH_3, the precursor of NH_4^+, with the rapid increase of the vehicle numbers. Previous studies indicated traffic source have become comparable to that of agriculture emission for NH_3 in 2013[18].

Factor 6 showed high loadings of Mn, Pb, Zn, Cu, Cd, and Fe, which were

treated as signals of industrial processes[6, 19]. Emissions from the iron and steel industry are possibly important among those industrial processes for two reasons. One is that the sintering process in the iron and steel industries emits large amounts of Pb, Hg, Zn and other heavy metal pollutants, and other processes such as ironmaking and steelmaking also emit fugitive dust containing high concentrations of heavy metals [5]. The other reason is the huge scale of steel production in North China. National statistical data shows that China produced approximately half the world's production of crude steel in 2014, and productions in the BTH region, Shandong province, and Liaoning province account for 27.9%, 7.2% and 8.7% of the total amount in China, respectively (available at the website http://www.stats.gov.cn/tjsj/ndsj/). Thus, iron and steel industries are likely the main atmospheric sources of the metal elements in this study. As shown in Figure S9, two peak values were found in August 31, 2012 and September 16, 2012, respectively. 72-h back trajectories indicate that the air mass on August 31, 2012 passed through the Shandong Peninsula before reaching the Tuoji Island (Figure S12). The air mass on September 16, 2012 passed Liaoning province before reaching the Tuoji Island (Figure S13). The two high contributions suggest their strong associated with distances between source regions and the sampling site.

Factor 7 was characterized by high loadings of SO_4^{2-}, NO_3^-, NH_4^+, Pb OC, and EC. Coal combustion is often indicated by elevated SO_4^{2-} linked with high OC and EC [6]. This source was the largest contributor of SO_4^{2-} in the present study, matching with the inventory results in North China[20]. Coal fly ash could be one of the main contributors of aerosol Pb in China as they contain abundant Pb and leaded gasoline was phased out nationwide in2000[21, 22]. For the source of NH_3, the precursor of NH_4^+, coal-fired power utilities equipped with selective catalytic reduction (SCR) and selective non-catalytic reduction (SNCR) technologies are significant source for "fuel NH_3". For example, based on nitrogen (N) isotopes analysis, a recent study reported that fossil fuel emission contributed 90% of the total NH_3 during the haze days in Beijing[23].

Factor 8 was characterized by high concentrations of K^+, OC, EC, SO_4^{2-} and NH_4^+. K^+ is extensively used as a tracer of biomass burning as mentioned above[24, 25]. Ratio of OC to EC from this source was the secondary highest (3.13) among the eight identified sources (0-3.72). The ratio was lower than that of mineral dust, suggesting mixing more OC from biogenic emission in mineral dust. In addition, high loading from the source was found on June 6, 2012, as shown in Figure S14. On the day, OC and K^+ concentrations were 14.4 μg m^{-3} and 4.37μg m^{-3}, respectively, reaching the highest concentrations during the entire sampling period. The air mass passed Shandong peninsula, when the extensive fire counts was found which was the typical characteristics of open burning of straw[26]. Thus, the factor represented emissions from biomass burning.

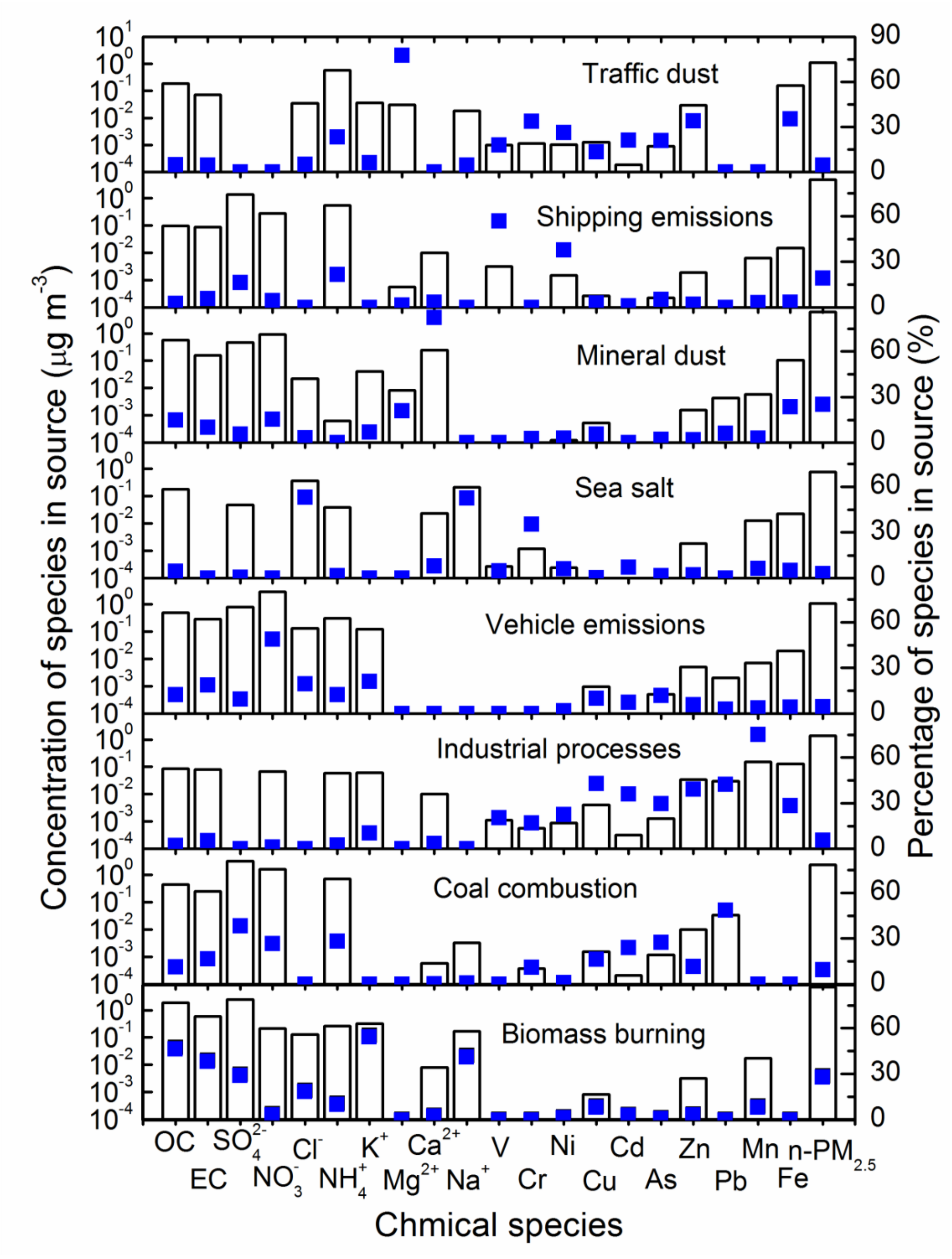

Figure S8. Concentration profiles and contributions of eight sources to $PM_{2.5}$ obtained from base model run of PMF. The concentration profile of each species is apportioned to the factor as a white bar corresponding to left y-axis with logarithmic scale and the percent of each species is apportioned to the factor as a blue box corresponding to right y-axis. The ordinal number of the factors from top to bottom was from first to eighth

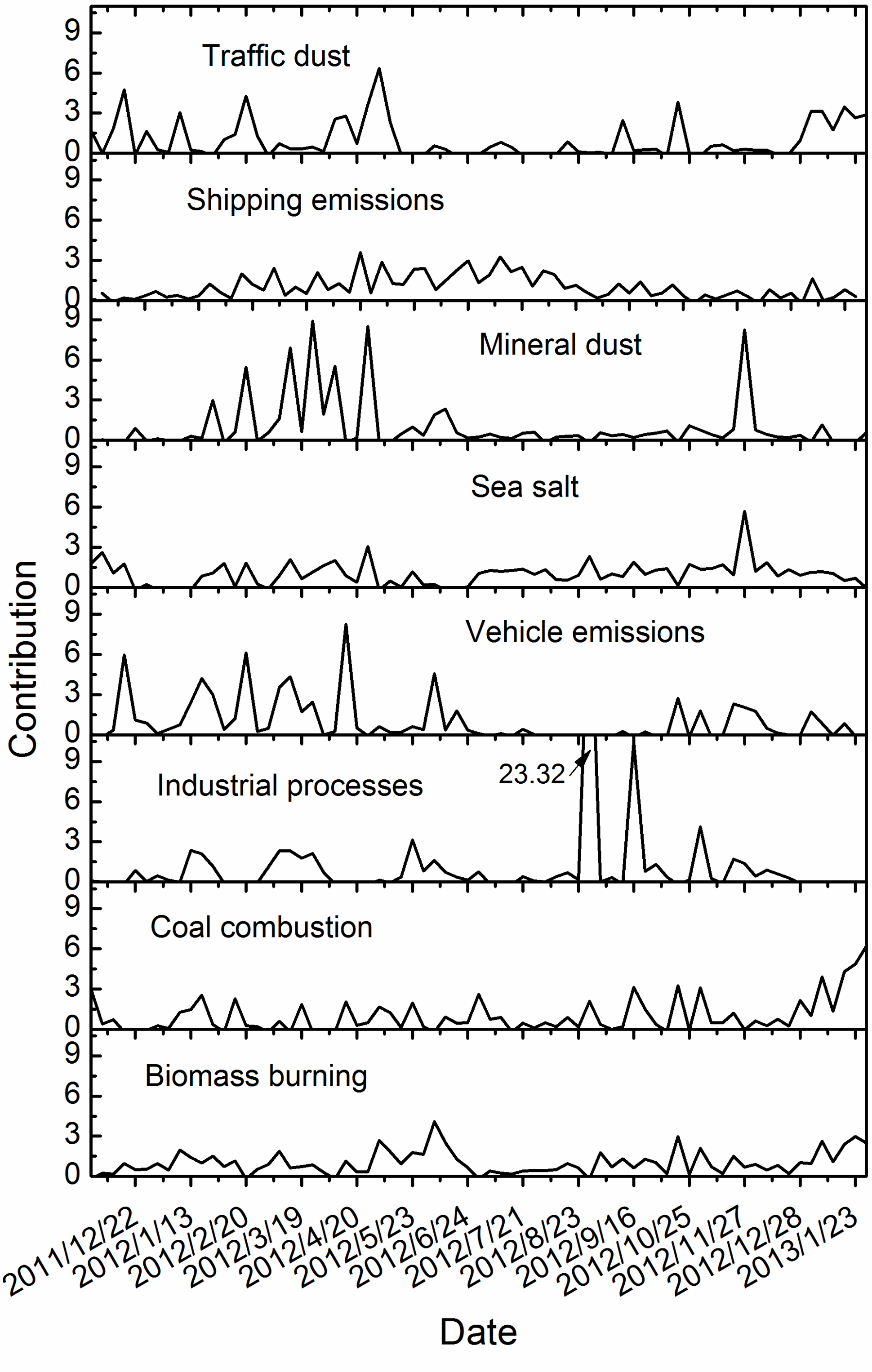

Figure S9. Time series of source contribution to the total mass obtained from PMF base model run with eight factors. The ordinal number of the factors from top to bottom was from first to eighth

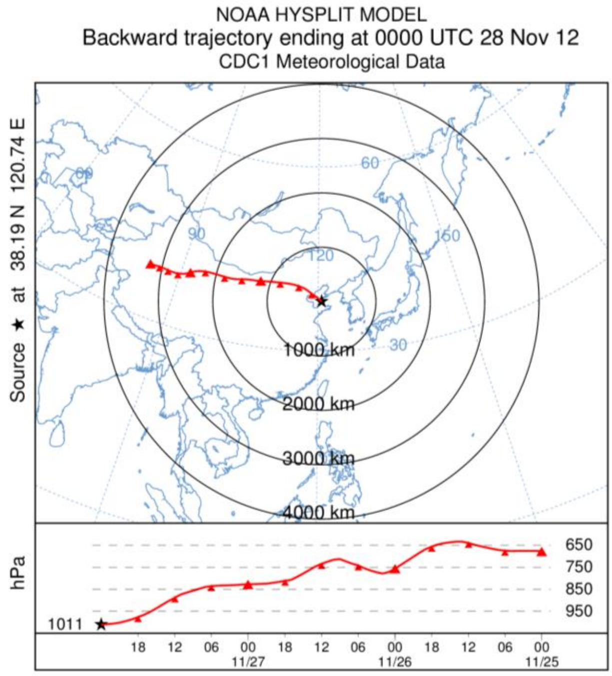

Figure S10. 72-h back trajectory of air masses at 00:00 UTC (coordinated universal time) on November 28, 2012 on Tuoji Island. The time is equivalent to 8:00 in local standard time (LST) on the same day. The trajectory is used to indicate movement of air mass from 10:00 a.m. on November 27 to 10:00 a.m. on November 28. The map was drawn by the software of HYSPLIT_win32U.exe (version 854), http://ready.arl.noaa.gov/HYSPLIT.php.

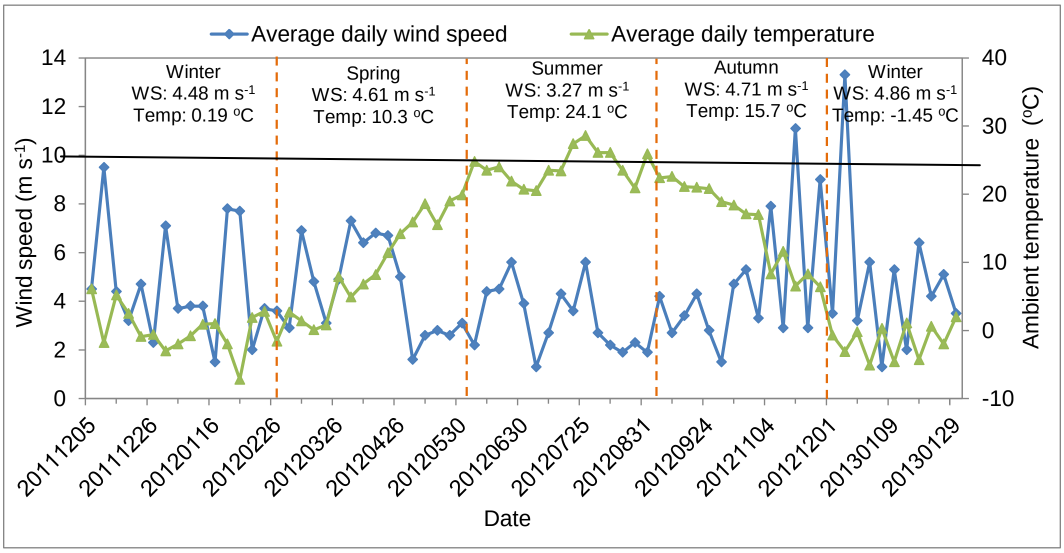

Figure S11. Avrages of daily wind speed and ambient temperature at Tuoji Island during the sampling period. The embedded WS and Temp indicate seasonal wind speed and ambient temperature, respectively

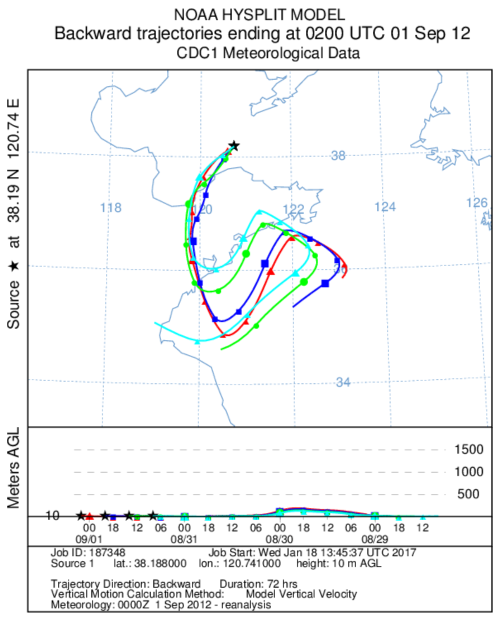

Figure S12. 72-h back trajectory of air masses at 02:00 UTC on September 1, 2012 at Tuoji Island. The four trajectories are used to indicate movement of air mass from 10:00, 16:00, 22:00 on August 31 and 02:00 on September 1. The map was drawn by the software of HYSPLIT_win32U.exe (version 854), http://ready.arl.noaa.gov/HYSPLIT.php.

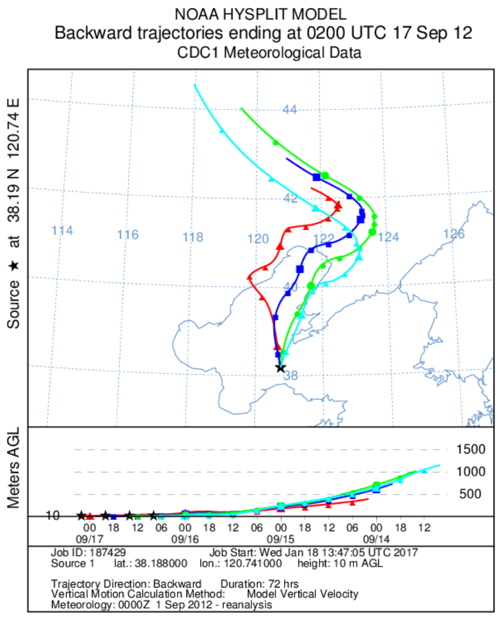

Figure S13. 72-h back trajectory of air masses at 02:00 UTC on September 17, 2012 at Tuoji Island. The four trajectories are used to indicate movement of air mass from 10:00, 16:00, 22:00 on September 16 to 02:00 on September 17. The map was drawn by the software of HYSPLIT_win32U.exe (version 854), http://ready.arl.noaa.gov/HYSPLIT.php.

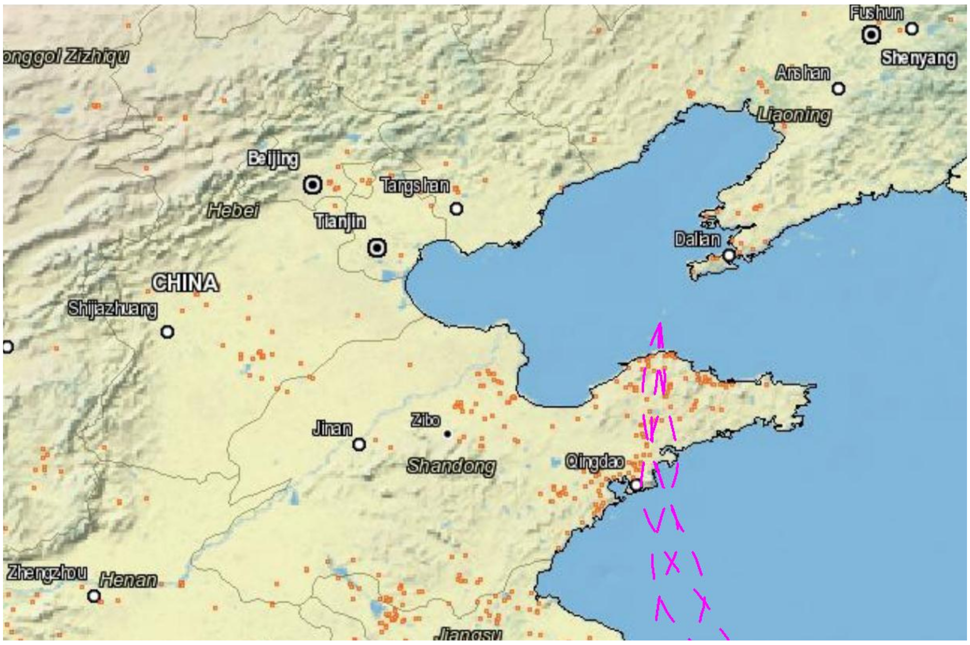

Figure S14. Accumulative satellite fire count (orange points) diagram during June 4 – 6 and 72-h back trajectories (pink dashed lines) on June 6, 2012. The map was drawn by the software of Surfer (version 9.0), http://www.goldensoftware.com/ and the basemap used was from the website of https://firms.modaps.eosdis.nasa.gov/firemap/.

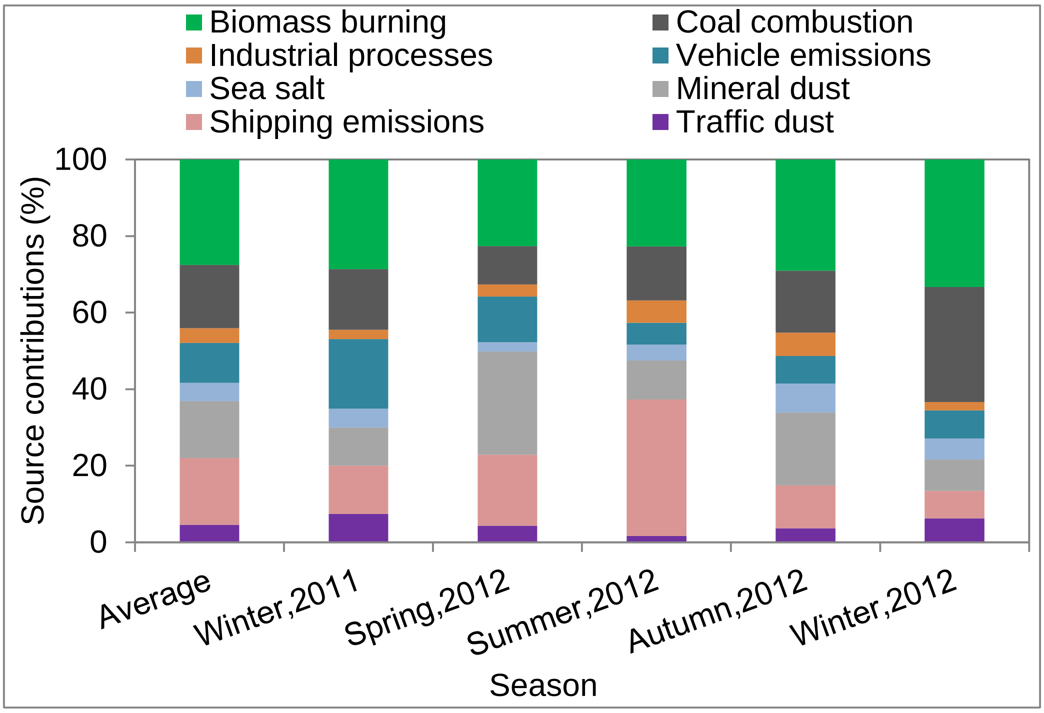

Figure S15. The contributions to $PM_{2.5}$ of eight sources identified by the PMF base model run

Text S3 Total and Seasonal Source Contributions of $PM_{2.5}$

Total and seasonal contributions (%) of the eight sources to $PM_{2.5}$ are shown in Figure S15. Among the eight sources, biomass burning, shipping emissions, and coal combustion were the largest contributors, which accounted for 27.5%, 17.5% and 16.5% of $PM_{2.5}$ mass concentrations during the entire sampling period, respectively. They were followed by mineral dust (14.8%), vehicle emissions (10.4%), sea salt (4.8%), traffic dust (4.6%), and industrial processes (3.9%).

During the winter of 2011, the contributions of biomass burning and coal combustion were comparable with that for the whole sampling period. The contributions of vehicle emissions and traffic dust to $PM_{2.5}$ were about 8% and 3% higher than the overall averages, respectively. The increases were mainly attributed to the outflow of airborne $PM_{2.5}$ from the Beijing-Tianjin-Hebei (BTH) region because the emission sector has been attracted the great concern in the region and prevailing northwestern wind dominated by the East Asian winter monsoon,[27] as shown the seasonal cluster back trajectories in Figure S16. Correspondingly, the contribution of shipping emissions was 5% lower than the average level, partly attributed strong signatures of land-sourced pollutants originated from the BTH region and partially because of weak navigation activity. Figure S17 shows monthly cargo throughput of ports around the Bohai Sea and its relative variation relative to the average during the entire sampling period. Cargo throughput in winter, 2011 was 6.7% lower than the average, which is comparable with the decrease of the contribution of shipping emissions to $PM_{2.5}$.

In the spring of 2012, the contribution of mineral dust to $PM_{2.5}$ increased sharply, up to 27% from 10% in the previous winter, which is in agreement with the high frequency of sandstorms in Hexi corridor and part of Inner Mongolia. The sandstorms can influence significantly on the part of the north China, including Bohai Sea [2]. During the season, the contribution of shipping emissions to $PM_{2.5}$ increased about 6% compared with that in the previous winter, and 1.0% compared with the

average of the whole sampling period. Correspondingly, the cargo throughput increased 8.5% and 1.8% relative to that in the previous winter and the average level. The coincident rise and decline of the contribution and cargo throughput, along with no obvious difference of wind trajectories in the first two seasons (Figure S17) indicate a strong correlation between ship activities and contribution of shipping emissions to $PM_{2.5}$.

The contribution of shipping emission increased continuously and reached 36% in summer when cargo throughput fell 2.5% than the average (Figure S17). The contributions of mineral dust, vehicle emission, and traffic dust decreased due to the air mass came mainly from the sea and low wind speed (Figures S10 and S11). During the autumn, the contributions of shipping emissions decreased sharply, from 36% in the previous summer to 11%. Except for the decline, contributions of other sources increased consistently, especially, mineral dust and biomass burning raised significantly, increased by 8.9% and 6.4% versus that in the previous season. The two increasing contributions to $PM_{2.5}$ were in agreement with that found in Beijing[6]. In the winter, the contribution of coal combustion increased sharply, reached to 30%, correspondingly, the contribution of biomass burning was up to 33%. Both the sources became dominant contributors to $PM_{2.5}$. The increase was which are attributed to addition consumption of the fuels for domestically heating.

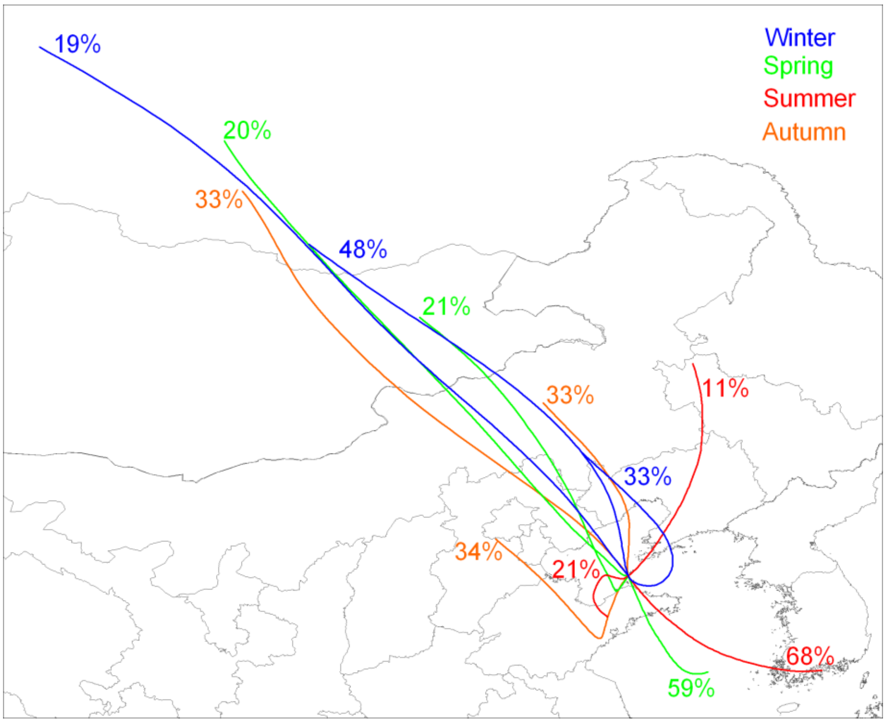

Figure S16. 72-h back trajectory cluster map for winter in 2011, spring, summer and autumn in 2012. The map was drawn by the software of Surfer (version 9.0), http://www.goldensoftware.com/

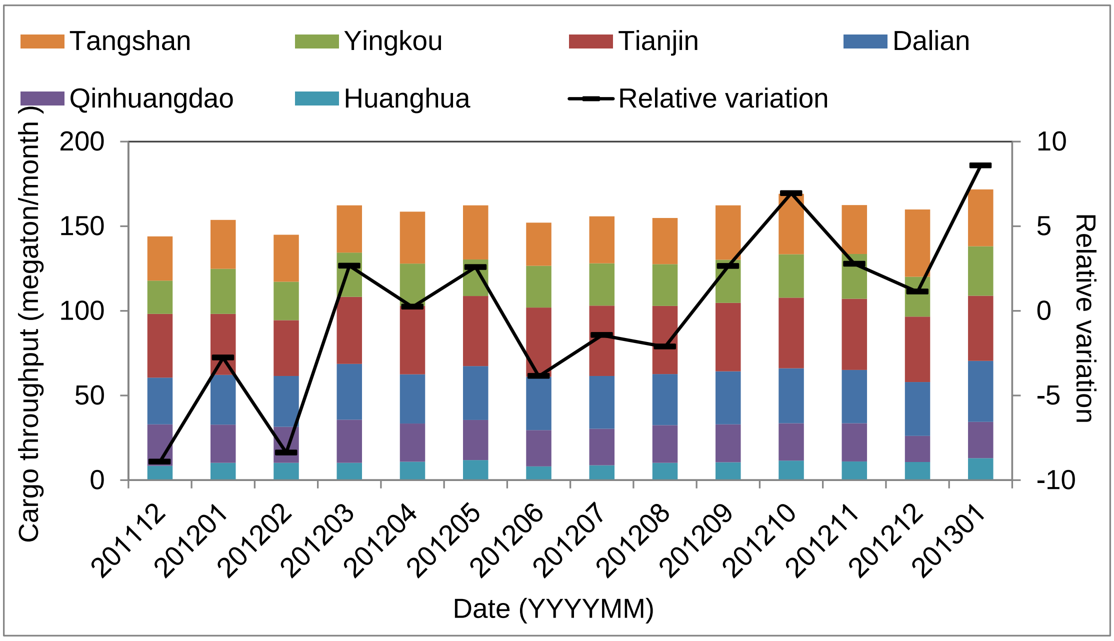

Figure S17. Monthly cargo throughput of ports around Bohai Sea and the relative variation of the monthly cargo throughput relative to average of the total cargo throughput during the whole sampling period. Note: the data were collected from website of China ports (http://www.chinaports.com)

Table S2. Distances between standardized source profiles from base run with seven and eight factors

	Traffic dust	Shipping emissions	Mineral dust	Sea salt	Vehicle emissions	Industrial processes	Coal combustion	Biomass burning
Traffic dust	1.26	6.19	6.91	6.18	7.16	6.54	6.59	7.57
Shipping emissions	6.66	1.13	6.94	6.75	6.93	6.81	6.61	6.04
Coal combustion& Biomass burning	7.77	6.85	6.84	7.11	6.07	7.02	4.21	3.15
Industrial processes	6.35	6.53	7.18	6.72	7.27	0.82	5.75	7.67
Mineral dust	6.23	6.44	0.97	6.67	7.09	6.98	7.32	6.71
Vehicle emissions	7.23	7.04	6.55	6.58	0.69	7.31	6.53	6.04
Sea salt	6.59	7.09	6.67	0.90	6.65	6.95	7.19	5.39

Table S3. Pearson correlation coefficient between time series of source contribution of base runs with seven and eight factors

	Traffic dust	Shipping emissions	Mineral dust	Sea salt	Vehicle emissions	Industrial processes	Coal combustion	Biomass burning
Traffic dust	0.97**	-0.06	-0.01	-0.06	0.31**	-0.22	0.48**	0.33**
Shipping emissions	-0.09	0.94**	-0.15	-0.35**	-0.15	-0.17	-0.17	0.10
Coal combustion& Biomass burning	0.31**	-0.14	-0.10	-0.25*	0.21	0.18	0.82**	0.79**
Industrial processes	-0.21	-0.09	-0.02	0.21	-0.04	0.99**	0.19	-0.14
Mineral dust	0.07	0.05	0.99**	0.51**	0.23	-0.01	-0.26	-0.12
Vehicle emissions	0.25	-0.09	0.43**	0.12	0.97**	-0.01	-0.08	0.22
Sea salt	-0.20	-0.30*	0.47**	0.88**	-0.10	0.12	-0.29*	-0.21

Note: The superscripts of '*' and '*' indicate the confidence level of 95 and 99%, respectively.

Table S4. Distances between standardized source profiles from base run with eight and nine factors

	Traffic dust	Shipping emissions	Mineral dust	Sea salt	Vehicle emissions	Industrial processes	Coal combustion	Biomass burning
Domestic coal	6.14	6.76	7.47	6.28	6.06	6.27	4.29	6.61
Industrial processes	6.20	6.23	7.08	6.77	7.08	2.16	6.94	7.15
Mineral dust & Sea salt	6.83	6.60	4.32	3.40	7.42	6.87	7.39	6.12
Shipping emissions	6.36	0.86	6.98	6.75	7.01	6.57	6.39	6.70
Industrial coal	6.73	6.86	6.73	6.49	6.80	5.24	4.08	6.97
Domestic biofuel	6.82	7.01	6.80	6.24	6.75	7.31	5.86	2.22
Traffic dust	2.12	6.31	6.19	6.15	6.71	6.95	7.57	7.20
Vehicle emissions	7.23	7.00	4.41	6.23	3.55	7.23	7.03	6.88
Open biomass burning	7.48	6.53	6.39	7.40	5.29	7.32	5.70	3.61

Table S5. Pearson correlation coefficient between time series of source contribution of base runs with eight and nine factors

	Traffic dust	Shipping emissions	Mineral dust	Sea salt	Vehicle emissions	Industrial processes	Coal combustion	Biomass burning
Domestic coal	0.77**	-0.10	-0.17	-0.10	.365**	0.00	0.75**	0.40**
Industrial processes	-0.22	-0.08	0.02	0.21	0.00	0.99**	0.15	-0.10
Mineral dust & Sea salt	-0.27*	0.00	0.56**	0.70**	-0.15	0.27*	-0.08	-0.09
Shipping emissions	-0.01	0.95**	-0.17	-0.35**	-0.16	-0.07	0.04	0.03
Industrial coal	-0.08	-0.10	-0.11	0.17	-0.07	0.92**	0.37**	-0.07
Domestic biofuel	0.06	-0.28	0.03	-0.11	0.01	-0.13	0.26*	0.60**
Traffic dust	0.87**	-0.02	0.45**	0.22	0.36**	-0.21	0.07	0.09
Vehicle emissions	-0.07	-0.02	0.68**	0.32**	0.58**	0.06	-0.22	0.04
Open biomass burning	0.23	0.16	0.23	-0.20	0.63**	0.03	0.27*	0.61**

Note: The superscripts of '*' and '*' indicate the confidence level of 95 and 99%, respectively.

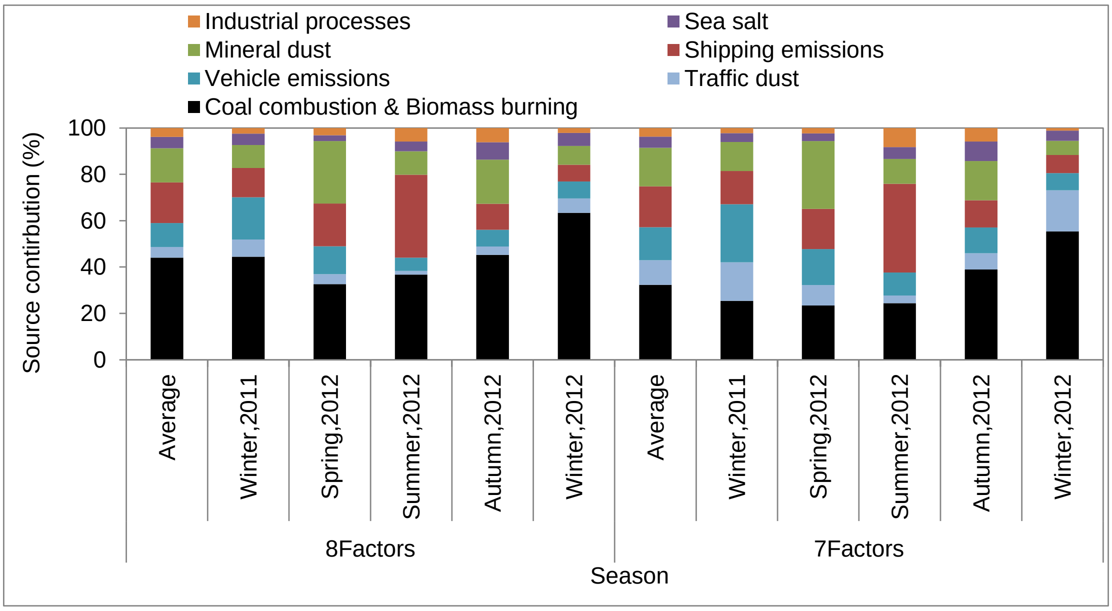

Figure S18. Comparison between source contributions to $PM_{2.5}$ from base model runs with eight and seven factors

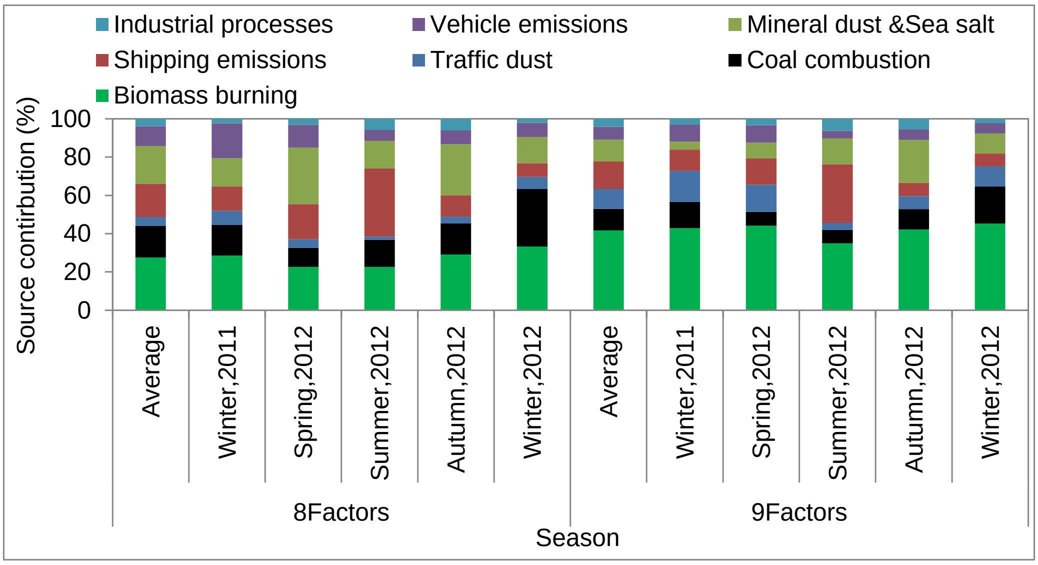

Figure S19. Comparison between source contributions to $PM_{2.5}$ from eight and nine factor base model runs

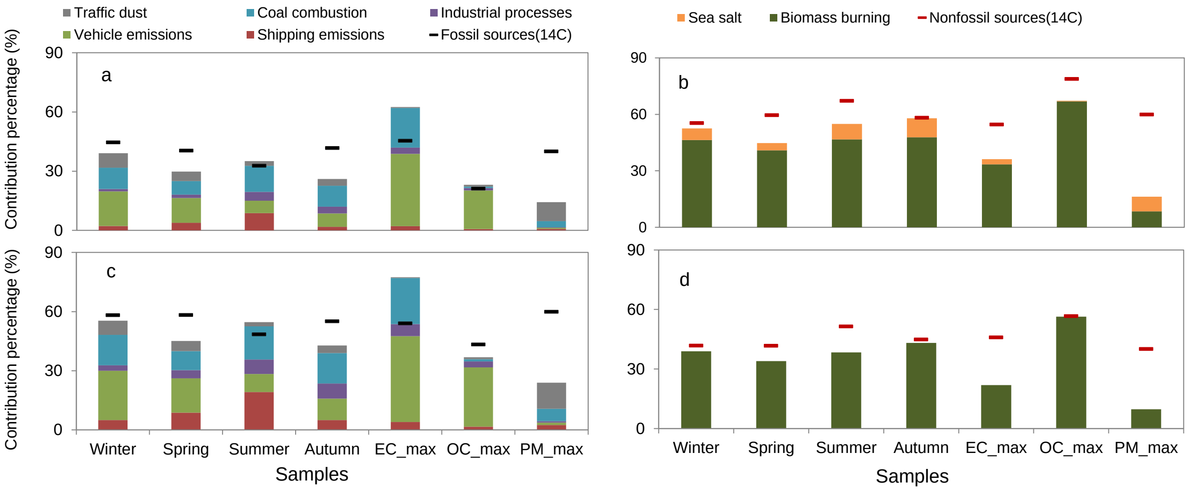

Figure S20. Comparison of the source apportionment of OC and EC classified from the PMF results and determined from the ^{14}C measurements. The PMF source classification was calculated based on equation (6) in the text. Biomass burning and sea salt identified by PMF were combined as a non-fossil source, while coal combustion, industrial processes, vehicle emissions, and shipping emissions were merged as fossil sources for the comparison. Mineral dust was not included and traffic dust was included as an additional fossil source in the comparison. Panels from a to d are the contributions to OC from fossil (a) and non-fossil (b) source, and to EC from fossil (c) and non-fossil source (d).

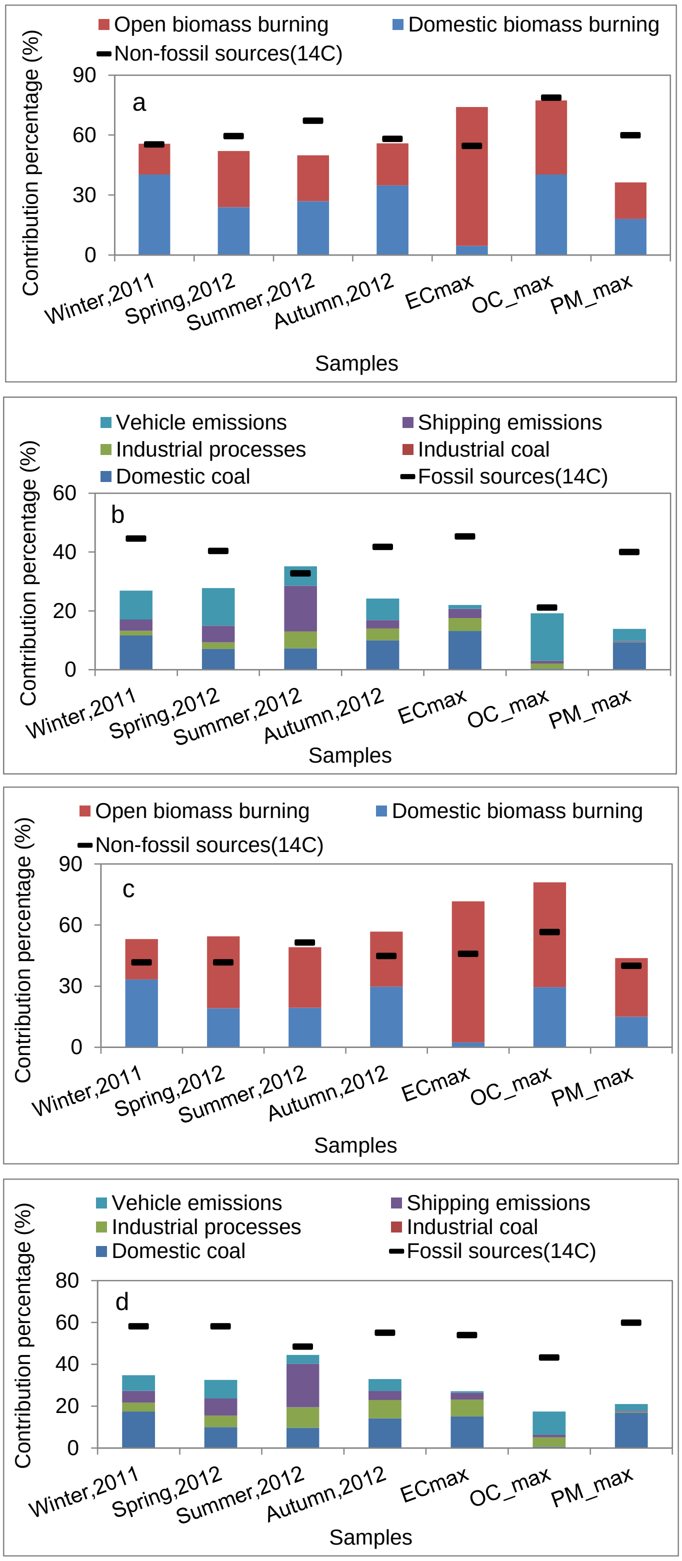

Figure S21. Source apportionments of OC and EC modeled by nine factor base model run and determined by ^{14}C results. Panels from a to d are the contributions to OC from non-fossil (a) and fossil (b) source, and to EC from non-fossil (c) and fossil source (d)

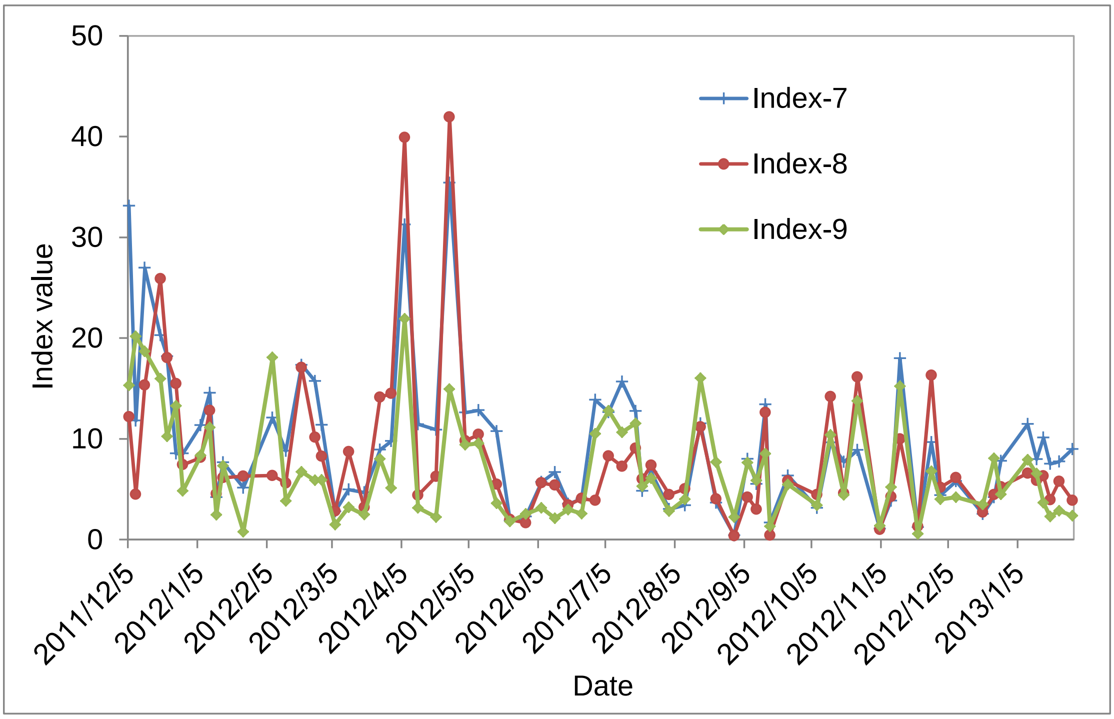

Figure S22. The developed index values (dimensionless) calculated by the data from base model runs with factors from seven to nine (BMR-7, BMR-8, and BMR-9)

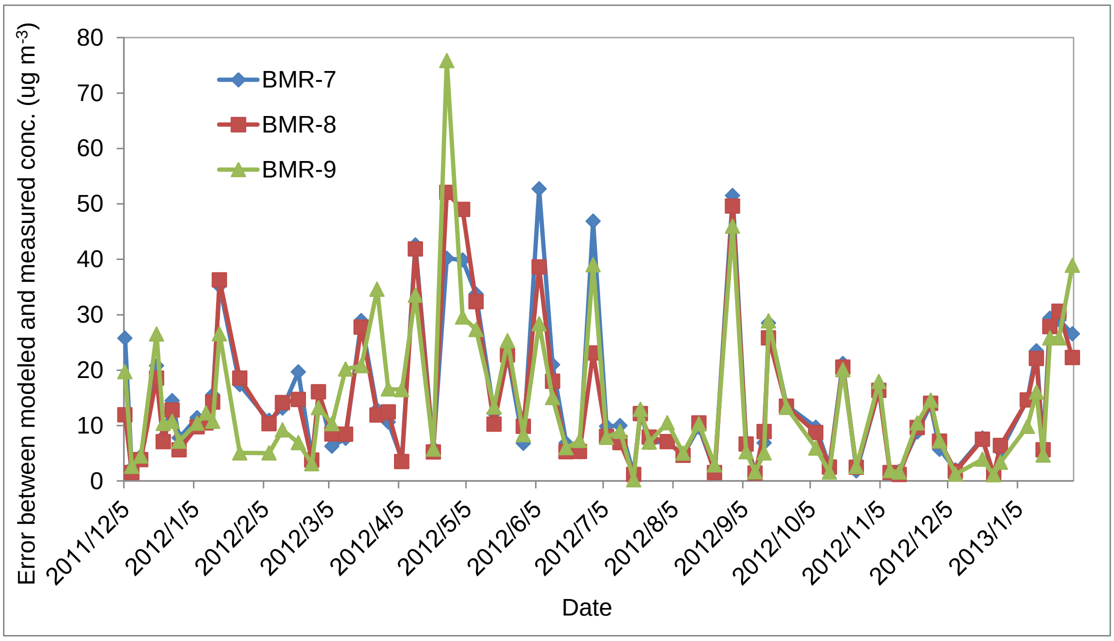

Figure S23. Errors between modeled by base model runs with factors from seven to nine and measured $PM_{2.5}$ concentrations (BMR-7, BMR-8, and BMR-9)

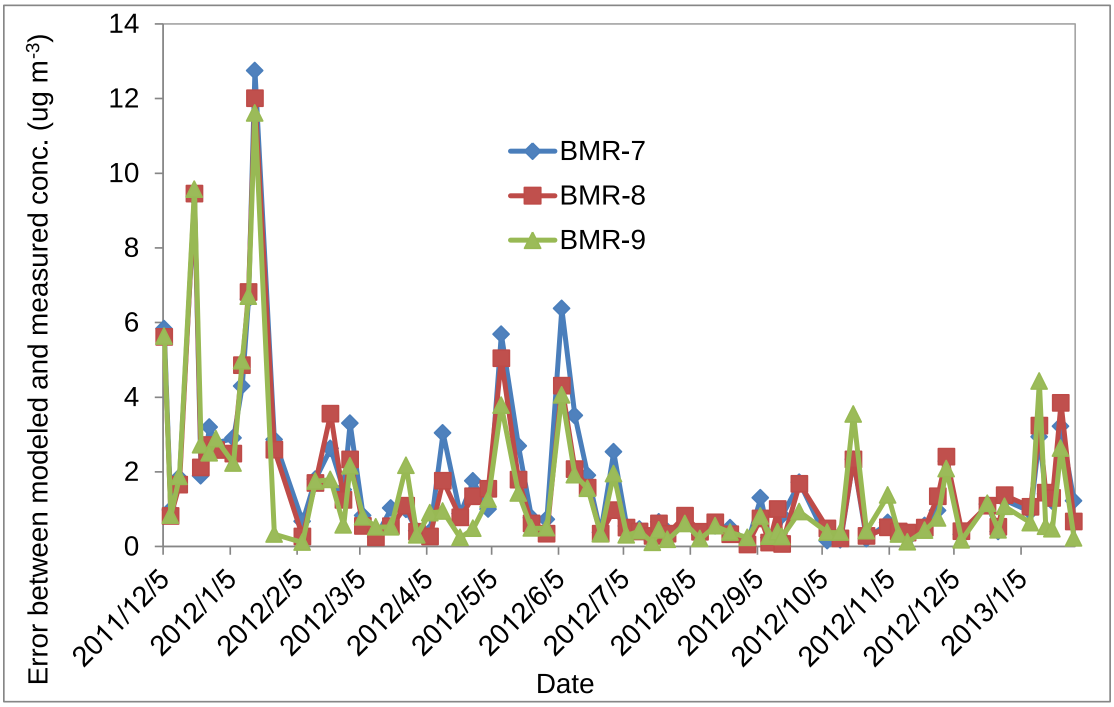

Figure S24. Errors between modeled by base model runs with factors from seven to nine and measured OC and EC concentrations (BMR-7, BMR-8, and BMR-9)

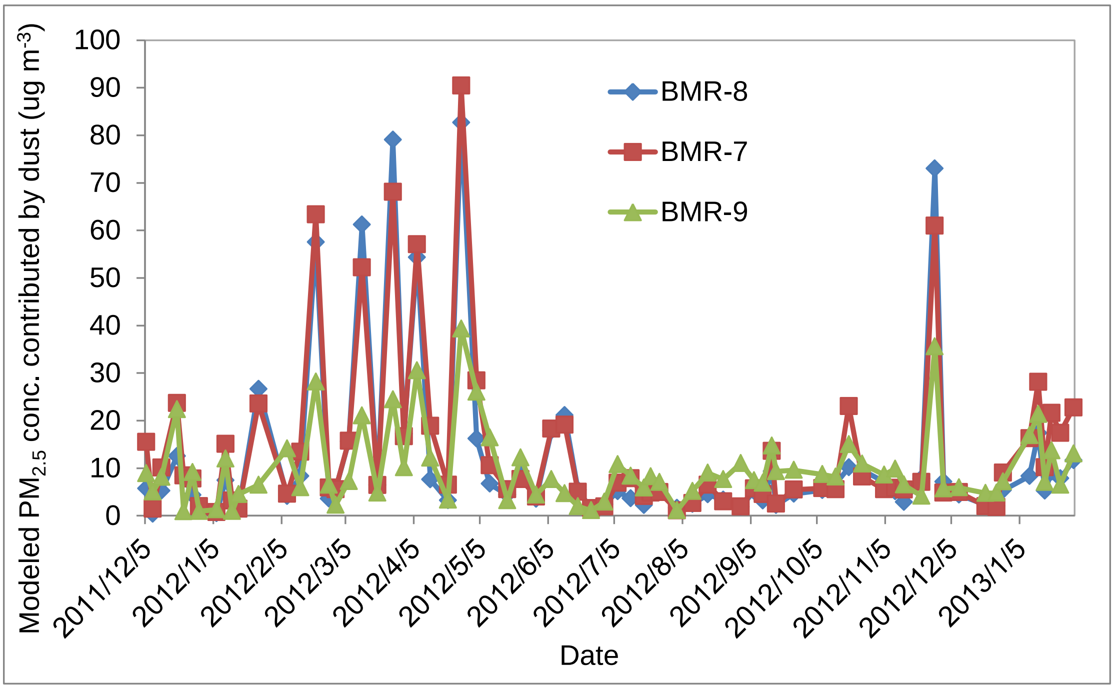

Figure S25. Modeled $PM_{2.5}$ concentrations contributed by dust sources from base model runs with factors from seven to nine (BMR-7, BMR-8, and BMR-9)

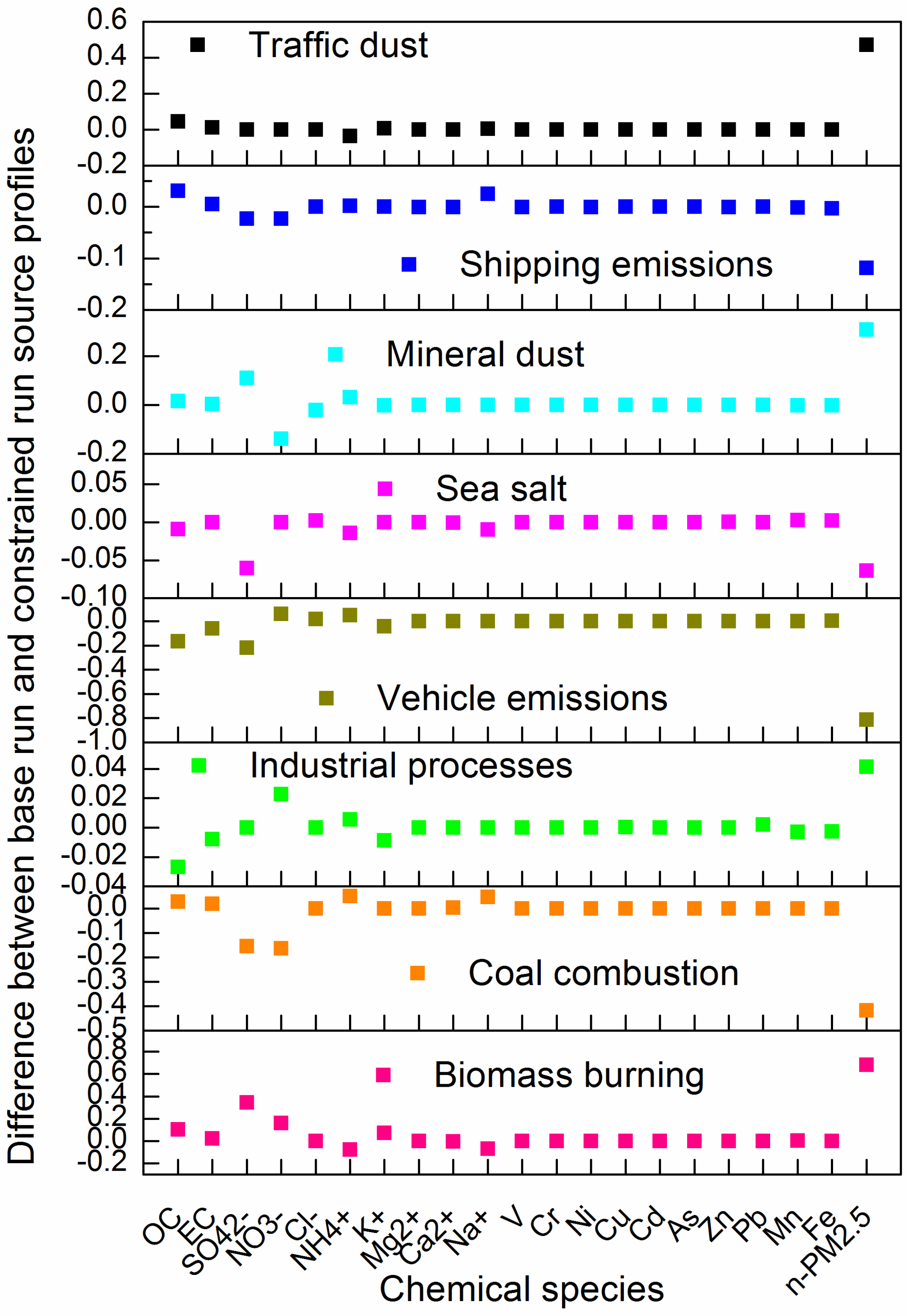

Figure S26. Differences of source profiles modeled by base model run and constrained model run with factor of eight

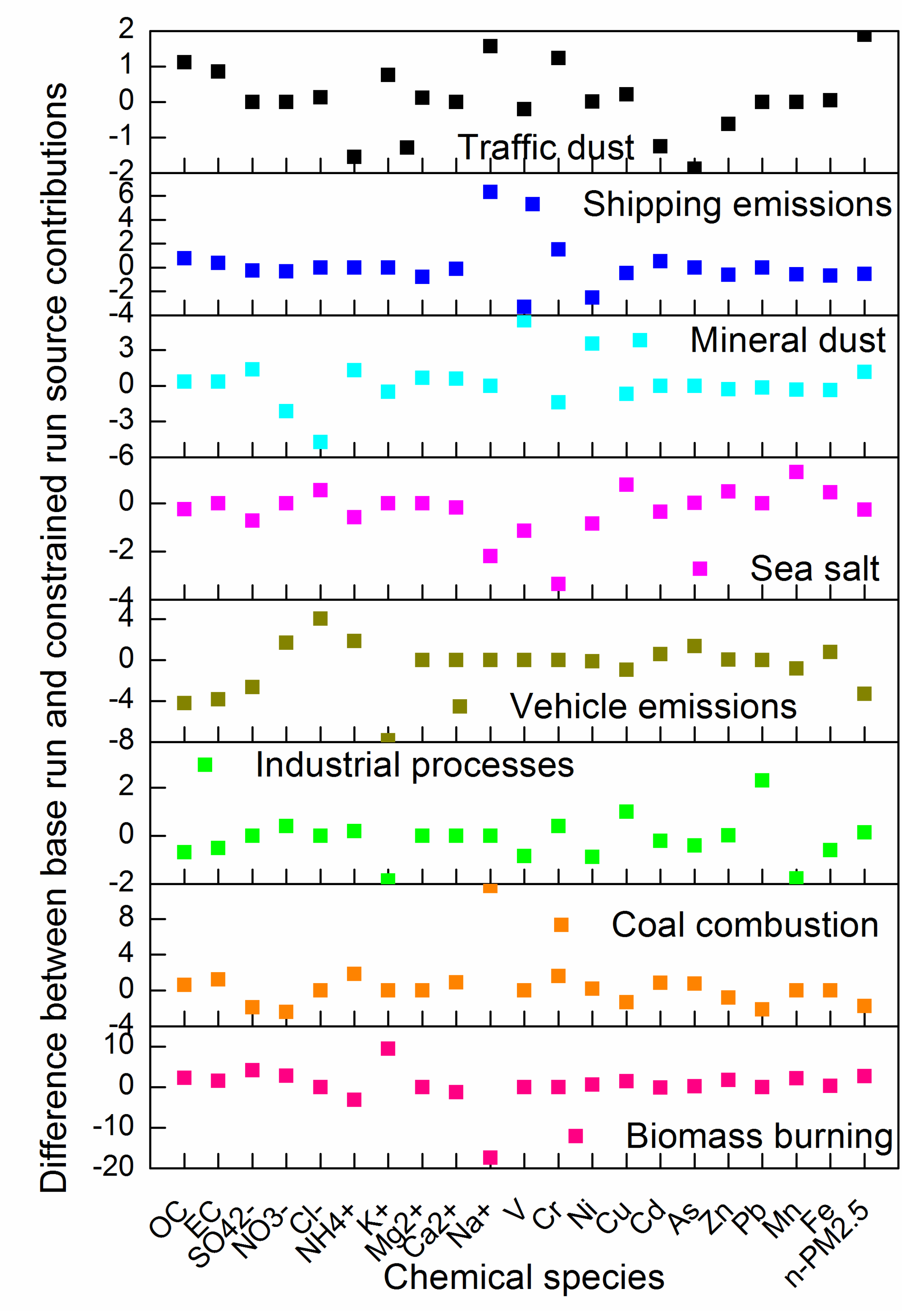

Figure S27. Differences of source contributions (%) modeled by base model run and constrained model run with factor of eight

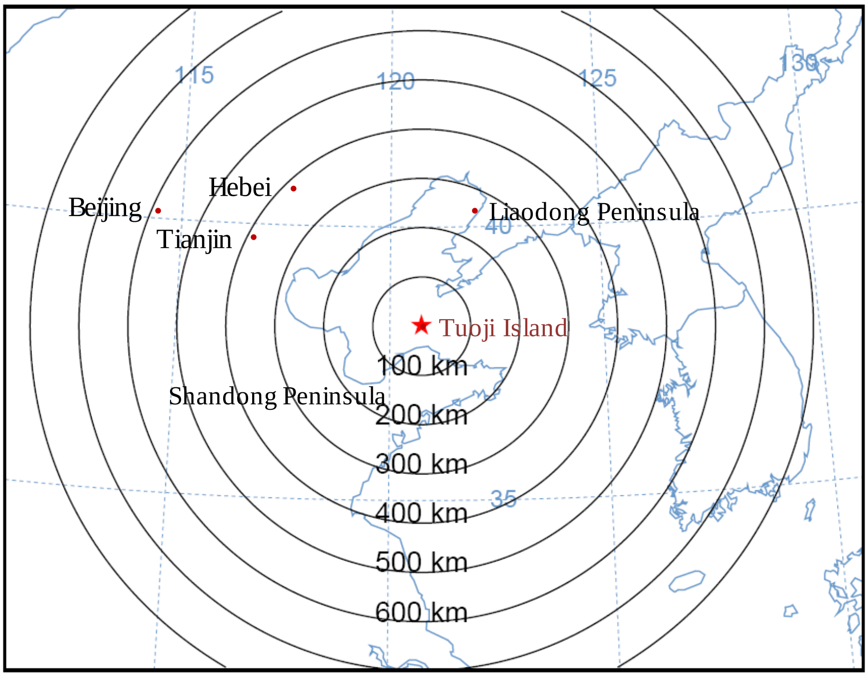

Figure S28. Distance between selected regions and the sampling site at Tuoji Island. The map was drawn by the software of HYSPLIT_win32U.exe (version 854), http://ready.arl.noaa.gov/HYSPLIT.php.

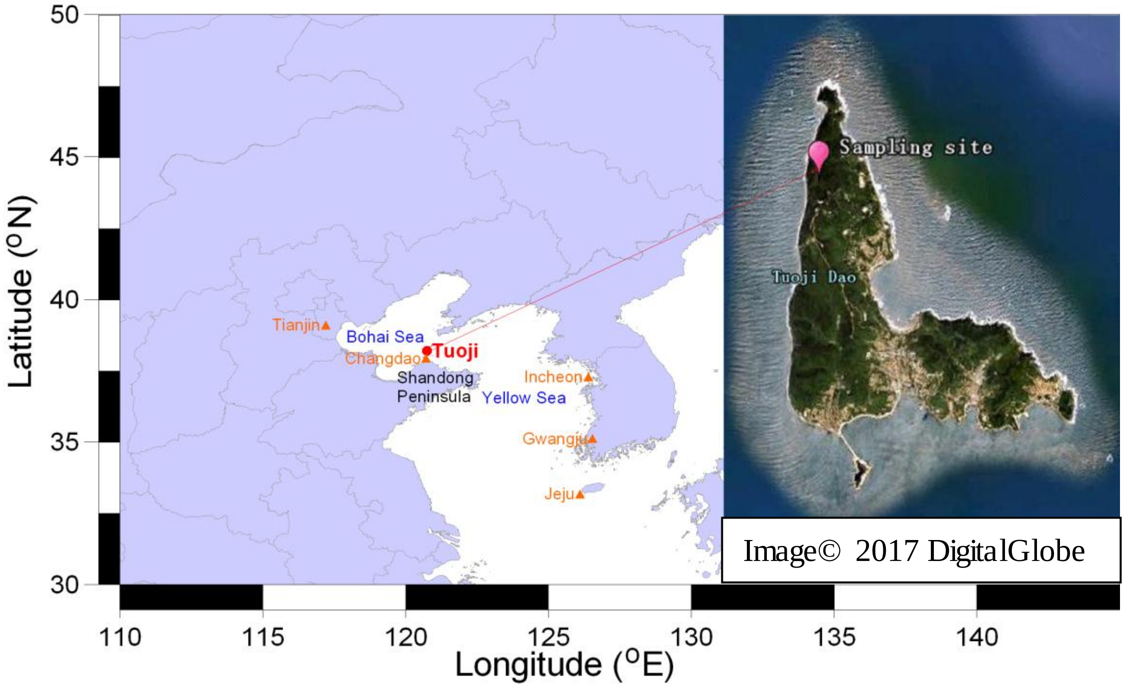

Figure S29. Map showing the sampling site. The site is located on the northwest coast of Tuoji Island, China. The map was drawn by the software of Surfer (version 9.0), http://www.goldensoftware.com/ and the embedded figure was generated by the software of Google Earth (version 7.1.7.2606), http://earth.google.com/

Text S4 Chemical Analysis

$PM_{2.5}$ mass concentration. Quartz fiber filters were analyzed gravimetrically for the mass concentrations of $PM_{2.5}$ using a Sartorius MC5 electronic microbalance with a ± 10 μg sensitivity. These filters were weighed after 24-h equilibration at temperature between 20 and 23°C and relative humidity between 35 and 45%. Each filter was weighed at least three times before and after sampling. The difference among the three repeated weighing was less than 10 μg for a blank filter and less than 20 μg for a sampling filter. The corresponding $PM_{2.5}$ mass concentration of each filter was equal to the weight difference before and after sampling divided by the sampled air volume.

OC and EC. After the measurement of $PM_{2.5}$ mass concentrations, organic carbon (OC) and elemental carbon (EC) were analyzed by a Desert Research Institute (DRI) Model 2001 Carbon analyzer (Atmoslytic Inc., Calabasas, CA) following the Interagency Monitoring of Protected Visual Environment (IMPROVE_A) thermal/optical reflectance (TOR) protocol[28]. A punch of 0.544 cm^2 from each quartz filter was heated to produce four fractions (OC1, OC2, OC3 and OC4) in four temperature steps (140, 280, 480, 580 ℃) under a non-oxidizing helium atmosphere and then in 2% O_2/98% He atmosphere at 580 ℃ (EC1), 740 ℃ (EC2), and 840 ℃ (EC3) for the EC fractions. At the same time, pyrolyzed organic carbon (POC) was produced in the inert atmosphere, which decreased the reflected light to correct for charred OC. The concentrations of OC and EC were obtained according to the IMPROVE protocol, OC = OC1 + OC2 + OC3 + OC4 + POC and EC = EC1 + EC2 +EC3 – POC. The detection limits of the method for OC and EC were 0.82 and 0.20 μg cm^{-2}, respectively. Blank filters and replicate samples were examined simultaneously after analyzing a batch of 10 samples to obtain their inherent concentrations on the filters and to evaluate measurement accuracy, respectively, using the same methods as described above. In this study, the contributions of OC and EC from blank filters were < 5.2 and 8.9% of their respective average concentrations. The uncertainties of OC (5.1%) and EC (6.3%) were calculated from the replicate

measurements.

Water-soluble ions. A 47 mm diameter punch was cut off from each quartz fiber filter, and the punch was ultra-extracted 15 min with 8 mL Milli-Q water (R>18.2 MΩ Millipore, Massachusetts, USA) for ionic measurement. The concentrations of water soluble ions: sodium (Na^+), ammonium (NH_4^+), potassium (K^+), magnesium (Mg^{2+}), calcium (Ca^{2+}), chlorine (Cl^-), nitrate (NO_3^-) and sulfate (SO_4^{2-}) were determined by ion chromatograph (Dionex ICS3000, Dionex Ltd., America) based on the measurement method of the reference[29]. 1 mL RbBr of 200 ppm was put in the solution as an internal standard before the analysis. The detection limit of water-soluble ions was 10 ng mL^{-1} with an error less than 5%.

Metal elements. A 47 mm diameter punch was digested with 5mL purified HNO_3 10 h at 120 ^oC for inorganic elemental measurement. The concentrations of metal elements: vanadium (V), manganese (Mn), iron (Fe), chromium (Cr), nickel (Ni), cuprum (Cu), zinc (Zn), arsenic (As), cadmium (Cd), and plumbum (Pb) were measured by an inductively coupled plasma mass spectrometry (ICP-MS of ELAN DRCII type, Perkin Elmer Ltd., Hong Kong) following the previous method[30]. Element indium (In) of 5 ppb was put in the solution as the internal standard before the analysis. The resolution of the ICP-MS ranged from 0.3 to 3.0 amu with a detection limit lower than 0.01 ng mL^{-1} and an error < 5%.

^{14}C measurement. ^{14}C measurement of OC and EC was performed by using an OC/EC separation system described in previous studies[31-36]. Firstly, OC was split into water-soluble organic carbon (WSOC) and water-insoluble organic carbon (WIOC) fractions. WSOC was extracted from a punch filter by Milli-Q water and was quantified as total dissolved organic carbon in solution by a total organic carbon (TOC) analyzer (Shimadzu TOC-VCPH, Japan). WIOC was quantified by OC given by the TOR protocol subtracting WSOC. As regards the WSOC, after being extracted by Milli-Q water, the extracted water was subsequently frozen in a 40 mL glass vial and freeze dried at -40 ℃ for 24-h. The residue was re-dissolved with 0.50 mL Milli-Q

water and subsequently transferred to a pre-combusted quartz tube, which was placed in the freeze dryer. The quartz tube was subsequently combusted at 850 ℃ and the carbon species were converted into CO_2. As regards the WIOC and the EC, after extraction, the filters were isolated at 340 ℃ for 15 min, after a flash heating of 650 ℃ for 45 s that could minimize charring. After the separation, the filters were removed from the system, placed into a muffle furnace heated to 375 ℃ and combusted for 4-h to remove the charring. Afterwards, the filters were quickly returned into the system and oxidized under a stream of pure oxygen at 650 ℃ for 10 min to analyze the EC fraction. Finally, the corresponding evolving CO_2 (WSOC, WIOC, and EC) was cryo-trapped, quantified manometrically, sealed in a quartz tube, and reduced to graphite at 600 ℃ using zinc with an iron (200 mg, Alfa Asear, 1.5–3 mm, 99.99%) catalyst for accelerator mass spectrometry (AMS) target preparation. The preparation of graphite targets was performed using the graphitization line at the Guangzhou Institute of Geochemistry, CAS. The ratios of $^{14}C/^{12}C$ in the graphite samples were determined through a NEC compact AMS at Peking University[37].

Generally, ^{14}C results were expressed as fractions of modern carbon (f_m), which is larger than 1 due to the nuclear bomb in 1950s and 1960s. It includes biogenic and biomass burning ($f_{m,bio}$, $f_{m,bb}$, respectively) and was estimated to be 1.06 ± 0.015 and 1.13 ± 0.05 for $f_{m,bio}$ and $f_{m,bb}$, respectively. Of them, $f_{m,bio}$ value was estimated from long term series of $^{14}CO_2$ measurement at Schauinsland station[38], while $f_{m,bb}$ was estimated by a tree-growth model[39]. In this study, f_m(EC) equals $f_{m,bb}$ assuming biomass burning is the only non-fossil source for EC, while f_m(OC) is adopted as the average value of $f_{m,bio}$ and $f_{m,bb}$ given OC originated equally from biogenic and biomass burning emission[33]. Finally, conversion factors were determined to be 1.10 and 1.06 for EC and OC, respectively, considering the steadily decline of ^{14}C after the factor estimation[37]. Thus, the fractions of non-fossil carbon (f_c) values in the samples were defined as $f_c = f_m/1.10$ for EC, $f_c = f_m/1.06$ for OC, and the fraction of fossil (f_f) was defined as $f_f = 1 - f_c$[31].

Text S5 Data Analysis

Backward Trajectories and Fire Counts. Backward trajectories and fire counts were used to assess potential sources of measured $PM_{2.5}$ in different air masses encountered during the sampling period. Backward trajectories were generated by the hybrid single-particle Lagrangian integrated trajectory (HYSPLIT) model, which is available on the National Oceanic and Atmospheric Administration Air Resource Laboratory website (www.arl.noaa.gov/ready/hysplit4.html)[40, 41]. Three day kinematic backward trajectories were generated at four different starting times during the 24 h sampling period with 6 h time intervals. The trajectories were calculated for air masses starting from the sampling site at 10 m above ground level using the six hourly objectively analyzed data from the United States National Center for Environmental Prediction (NCEP) reanalysis. Thus, four backward trajectories were generated for each sample. A total of 280 trajectories were generated and these trajectories were bunched into three clusters by the clustering function in the HYSPLIT model. Fire counts were detected by MODIS (Moderate Resolution Imaging Spectroradiometer) on the NASA satellites and the integrated data are available at http://firms.modaps.eosdis.nasa.gov/firemap/. One fire count in the map represents an active fire in a resolution of 1 × 1 km pixel.

Distance Analysis and Pearson Correlation. Based on the eight kind sources identified by the base model run with eight factors, source types modeled by the base model run with seven and nine factors were quickly determined by the distance analysis and the Pearson correlation. Firstly, all source profiles were standardized by their respective standard deviation, and then Euclidean distances between each of seven/nine source profiles and every one among the eight source factors were calculated by following equation:

$$D_{ij} = \sqrt{\sum_{k=1}^{22} \left(Y_{ik} - Z_{jk}\right)^2} \quad (2)$$

where D_{ij} is distance between i^{th} source profile of the base run (Y) with seven or nine

factors and j^{th} source profile (Z) of the eight factor base run. For a source factor from the base model run with eight factors, there are seven or nine distances for the base model run of the corresponding factor numbers. A factor with the shortest distance among the seven or nine distances has the greatest similar patterns with the source factor type of the base model run with eight factors. Time series of source contribution of seven and nine factor base model run were correlated (Pearson correlation) with that of eight factor base model run, respectively. Similar to the distance analysis, a factor with the highest correlation coefficient among the seven or nine correlation coefficients has the greatest similar patterns with the source type of the base model run with eight factors. The distance analysis and correlation evaluation were performed by SPSS v17.0.

REFERENCE

1. Li, J.; Song, Y.; Mao, Y.; Mao, Z.; Wu, Y.; Li, M.; Huang, X.; He, Q.; Hu, M., Chemical characteristics and source apportionment of $PM_{2.5}$ during the harvest season in eastern China's agricultural regions. *Atmospheric Environment* **2014,** *92*, (0), 442-448.

2. Tan, S.-C.; Shi, G.-Y.; Wang, H., Long-range transport of spring dust storms in Inner Mongolia and impact on the China seas. *Atmospheric Environment* **2012,** *46*, (0), 299-308.

3. Jeong, G. Y.; Kim, J. Y.; Seo, J.; Kim, G. M.; Jin, H. C.; Chun, Y., Long-range transport of giant particles in Asian dust identified by physical, mineralogical, and meteorological analysis. *Atmos. Chem. Phys. Discuss.* **2014,** *14*, (1), 505-521.

4. Ni, T.; Li, P.; Han, B.; Bai, Z.; Ding, X.; Wang, Q.; Huo, J.; Lu, B., Spatial and temporal variation of chemical composition and mass closure of ambient PM_{10} in Tianjin, China. *Aerosol Air Qual. Res.* **2013,** *13*, (6), 1832-1846.

5. Duan, J.; Tan, J., Atmospheric heavy metals and Arsenic in China: Situation, sources and control policies. *Atmospheric Environment* **2013,** *74*, (0), 93-101.

6. Zhang, R.; Jing, J.; Tao, J.; Hsu, S. C.; Wang, G.; Cao, J.; Lee, C. S. L.; Zhu, L.; Chen, Z.; Zhao, Y.; Shen, Z., Chemical characterization and source apportionment of $PM_{2.5}$ in Beijing: seasonal perspective. *Atmos. Chem. Phys. Discuss.* **2013,** *13*, (14), 7053-7074.

7. Cappa, C. D.; Williams, E. J.; Lack, D. A.; Buffaloe, G. M.; Coffman, D.; Hayden, K. L.; Herndon, S. C.; Lerner, B. M.; Li, S. M.; Massoli, P.; McLaren, R.; Nuaaman, I.; Onasch, T. B.; Quinn, P. K., A case study into the measurement of ship emissions from plume intercepts of the NOAA ship Miller Freeman. *Atmos. Chem. Phys. Discuss.* **2014,** *14*, (3), 1337-1352.

8. Pey, J.; Pérez, N.; Cortés, J.; Alastuey, A.; Querol, X., Chemical fingerprint and impact of shipping emissions over a western Mediterranean metropolis: Primary and aged contributions. *Science of The Total Environment* **2013,** *463-464*, (0), 497-507.

9. Zhang, F.; Chen, Y.; Tian, C.; Wang, X.; Huang, G.; Fang, Y.; Zong, Z., Identification and quantification of shipping emissions in Bohai Rim, China. *Science of The Total Environment* **2014,** *497–498*, (0), 570-577.

10. Gupta, D.; Kim, H.; Park, G.; Li, X.; Eom, H. J.; Ro, C. U., Hygroscopic properties of NaCl and NaNO3 mixture particles as reacted inorganic sea-salt aerosol surrogates. *Atmos. Chem. Phys. Discuss.* **2015,** *15*, (6), 3379-3393.

11. Wilson, T. W.; Ladino, L. A.; Alpert, P. A.; Breckels, M. N.; Brooks, I. M.; Browse, J.; Burrows, S. M.; Carslaw, K. S.; Huffman, J. A.; Judd, C.; Kilthau, W. P.; Mason, R. H.; McFiggans, G.; Miller, L. A.; Najera, J. J.; Polishchuk, E.; Rae, S.; Schiller, C. L.; Si, M.; Temprado, J. V.; Whale, T. F.; Wong, J. P. S.; Wurl, O.; Yakobi-Hancock, J. D.; Abbatt, J. P. D.; Aller, J. Y.; Bertram, A. K.; Knopf, D. A.; Murray, B. J., A marine biogenic source of atmospheric ice-nucleating particles. *Nature* **2015,** *525*, (7568), 234-238.

12. Chang, Y.; Zou, Z.; Deng, C.; Huang, K.; Collett, J. L.; Lin, J.; Zhuang, G., The importance of vehicle emissions as a source of atmospheric ammonia in the megacity of Shanghai. *Atmos. Chem. Phys. Discuss.* **2016,** *16*, (5), 3577-3594.

13. Tan, J.-H.; Duan, J.-C.; Ma, Y.-L.; Yang, F.-M.; Cheng, Y.; He, K.-B.; Yu, Y.-C.; Wang, J.-W., Source of atmospheric heavy metals in winter in Foshan, China. *Science of the Total Environment* **2014,** *493*, 262-270.

14. Jing, B.; Wu, L.; Mao, H.; Gong, S.; He, J.; Zou, C.; Song, G.; Li, X.; Wu, Z., Development of a vehicle emission inventory with high temporal-spatial resolution based on NRT traffic data and its impact on air pollution in Beijing - Part 1: Development and evaluation of vehicle emission inventory. *Atmos. Chem. Phys. Discuss.* **2016,** *16*, (5), 3161-3170.

15. Zhang, S.; Wu, Y.; Wu, X.; Li, M.; Ge, Y.; Liang, B.; Xu, Y.; Zhou, Y.; Liu, H.; Fu, L.; Hao, J., Historic and future trends of vehicle emissions in Beijing, 1998–2020: A policy assessment for the most stringent vehicle emission control program in China. *Atmospheric Environment* **2014,** *89*, (0), 216-229.

16. Shen, X.; Yao, Z.; Huo, H.; He, K.; Zhang, Y.; Liu, H.; Ye, Y., PM2.5 emissions from light-duty gasoline vehicles in Beijing, China. *Science of The Total Environment* **2014,** *487*, 521-527.

17. Shi, Y.; Xia, Y.-f.; Lu, B.-h.; Liu, N.; Zhang, L.; Li, S.-j.; Li, W., Emission inventory and trends of NOx for China, 2000–2020. *J. Zheijang Univ.-Sci.* **2014,** *15*, (6), 454-464.

18. Ianniello, A.; Spataro, F.; Esposito, G.; Allegrini, I.; Hu, M.; Zhu, T., Chemical characteristics of inorganic ammonium salts in PM2.5 in the atmosphere of Beijing (China). *Atmos. Chem. Phys. Discuss.* **2011,** *11*, (21), 10803-10822.

19. Amil, N.; Latif, M. T.; Khan, M. F.; Mohamad, M., Seasonal variability of PM2.5 composition and sources in the Klang Valley urban-industrial environment. *Atmos. Chem. Phys. Discuss.* **2016,** *16*, (8), 5357-5381.

20. Zhao, B.; Wang, P.; Ma, J. Z.; Zhu, S.; Pozzer, A.; Li, W., A high-resolution emission inventory of primary pollutants for the Huabei region, China. *Atmos. Chem. Phys. Discuss.* **2012,** *12*, (1), 481-501.

21. Cheng, H.; Hu, Y., Lead (Pb) isotopic fingerprinting and its applications in lead

pollution studies in China: A review. *Environmental Pollution* **2010,** *158,* (5), 1134-1146.

22. Zhang, Y.; Wang, X.; Chen, H.; Yang, X.; Chen, J.; Allen, J. O., Source apportionment of lead-containing aerosol particles in Shanghai using single particle mass spectrometry. *Chemosphere* **2009,** *74,* (4), 501-507.

23. Pan, Y.; Tian, S.; Liu, D.; Fang, Y.; Zhu, X.; Zhang, Q.; Zheng, B.; Michalski, G.; Wang, Y., Fossil fuel combustion-related emissions dominate atmospheric ammonia sources during severe haze episodes: Evidence from ^{15}N-stable isotope in size-resolved aerosol ammonium. *Environmental Science & Technology* **2016,** *50,* (15), 8049–8056.

24. Tao, J.; Zhang, L.; Zhang, R.; Wu, Y.; Zhang, Z.; Zhang, X.; Tang, Y.; Cao, J.; Zhang, Y., Uncertainty assessment of source attribution of $PM_{2.5}$ and its water-soluble organic carbon content using different biomass burning tracers in positive matrix factorization analysis — A case study in Beijing, China. *Science of The Total Environment* **2016,** *543, Part A,* 326-335.

25. Zhang, Y.; Obrist, D.; Zielinska, B.; Gertler, A., Particulate emissions from different types of biomass burning. *Atmospheric Environment* **2013,** *72,* (0), 27-35.

26. Wang, X.; Chen, Y.; Tian, C.; Huang, G.; Fang, Y.; Zhang, F.; Zong, Z.; Li, J.; Zhang, G., Impact of agricultural waste burning in the Shandong Peninsula on carbonaceous aerosols in the Bohai Rim, China. *Science of the Total Environment* **2014,** *481,* (0), 311-316.

27. Feng, J.; Guo, Z.; Chan, C. K.; Fang, M., Properties of organic matter in $PM_{2.5}$ at Changdao Island, China—A rural site in the transport path of the Asian continental outflow. *Atmospheric Environment* **2007,** *41,* (9), 1924-1935.

28. Chow, J. C.; Watson, J. G.; Chen, L. W. A.; Chang, M. C. O.; Robinson, N. F.; Trimble, D.; Kohl, S., The IMPROVE_A temperature protocol for thermal/optical carbon analysis: Maintaining consistency with a long-term database. *Journal of the Air & Waste Management Association* **2007,** *57,* (9), 1014-1023.

29. Shahsavani, A.; Naddafi, K.; Jaafarzadeh Haghighifard, N.; Mesdaghinia, A.; Yunesian, M.; Nabizadeh, R.; Arhami, M.; Yarahmadi, M.; Sowlat, M. H.; Ghani, M.; Jonidi Jafari, A.; Alimohamadi, M.; Motevalian, S. A.; Soleimani, Z., Characterization of ionic composition of TSP and PM_{10} during the Middle Eastern Dust (MED) storms in Ahvaz, Iran. *Environ Monit Assess* **2012,** *184,* (11), 6683-6692.

30. Wang, X.; Bi, X.; Sheng, G.; Fu, J., Hospital indoor PM_{10}/$PM_{2.5}$ and associated trace elements in Guangzhou, China. *Science of The Total Environment* **2006,** *366,* (1), 124-135.

31. Zong, Z.; Chen, Y.; Tian, C.; Fang, Y.; Wang, X.; Huang, G.; Zhang, F.; Li, J.; Zhang, G., Radiocarbon-based impact assessment of open biomass burning on regional carbonaceous aerosols in North China. *Science of The Total Environment* **2015,** *518–519,* (0), 1-7.

32. Liu, D.; Li, J.; Zhang, Y.; Xu, Y.; Liu, X.; Ding, P.; Shen, C.; Chen, Y.; Tian, C.; Zhang, G., The use of levoglucosan and radiocarbon for source apportionment of $PM_{2.5}$ carbonaceous aerosols at a background site in East China. *Environmental Science & Technology* **2013,** *47,* (18), 10454-10461.

33. Zhang, Y.-L.; Li, J.; Zhang, G.; Zotter, P.; Huang, R.-J.; Tang, J.-H.; Wacker, L.; Prévôt, A. S. H.; Szidat, S., Radiocarbon-based source apportionment of carbonaceous aerosols at a regional background site on Hainan Island, South China. *Environmental Science & Technology* **2014,** *48,* (5), 2651-2659.

34. Xu, X.; Trumbore, S. E.; Zheng, S.; Southon, J. R.; McDuffee, K. E.; Luttgen, M.; Liu, J. C., Modifying a sealed tube zinc reduction method for preparation of AMS graphite targets: Reducing background and attaining high precision. *Nuclear Instruments and Methods in Physics Research Section B: Beam Interactions with Materials and Atoms* **2007,** *259,* (1), 320-329.

35. Zhang, Y. L.; Liu, D.; Shen, C. D.; Ding, P.; Zhang, G., Development of a preparation system for the radiocarbon analysis of organic carbon in carbonaceous aerosols in China. *Nuclear Instruments and Methods in Physics Research Section B:*

Beam Interactions with Materials and Atoms **2010,** *268*, (17–18), 2831-2834.

36. Wacker, L.; Fahrni, S. M.; Hajdas, I.; Molnar, M.; Synal, H. A.; Szidat, S.; Zhang, Y. L., A versatile gas interface for routine radiocarbon analysis with a gas ion source. *Nuclear Instruments and Methods in Physics Research Section B: Beam Interactions with Materials and Atoms* **2013,** *294*, 315-319.

37. Liu, J.; Li, J.; Zhang, Y.; Liu, D.; Ding, P.; Shen, C.; Shen, K.; He, Q.; Ding, X.; Wang, X.; Chen, D.; Szidat, S.; Zhang, G., Source apportionment using radiocarbon and organic tracers for $PM_{2.5}$ carbonaceous aerosols in Guangzhou, South China: Contrasting local- and regional-scale haze events. *Environmental Science & Technology* **2014,** *48*, (20), 12002-12011.

38. Levin, I.; Naegler, T.; Kromer, B.; Diehl, M.; Francey, R. J.; Gomez-Pelaez, A. J.; Steele, L. P.; Wagenbach, D.; Weller, R.; Worthy, D. E., Observations and modelling of the global distribution and long-term trend of atmospheric $^{14}CO_2$. *Tellus Series B-Chemical and Physical Meteorology* **2010,** *62*, (1), 26-46.

39. Mohn, J.; Szidat, S.; Fellner, J.; Rechberger, H.; Quartier, R.; Buchmann, B.; Emmenegger, L., Determination of biogenic and fossil CO_2 emitted by waste incineration based on (CO_2)-C-14 and mass balances. *Bioresource Technology* **2008,** *99*, (14), 6471-6479.

40. Draxler, R. R.; Rolph, G. D., HYSPLIT (HYbrid Single-Particle Lagrangian Integrated Trajectory) Model access via NOAA ARL READY Website (http://www.arl.noaa.gov/ready/hysplit4.html). *Silver Spring, MD* **2003**.

41. Stein, A. F.; Draxler, R. R.; Rolph, G. D.; Stunder, B. J. B.; Cohen, D. D.; Ngan, F., NOAA's HYSPLIT Atmospheric Transport and Dispersion Modeling System. *Bulletin of the American Meteorological Society* **2015,** *96*, (12), 2059-2077.
